# Shifts in Chromosome Evolution Rates Shape the Karyotype Patterns of Leafcutting Ants

**DOI:** 10.1002/ece3.70602

**Published:** 2024-11-21

**Authors:** Danon Clemes Cardoso, Maykon Passos Cristiano

**Affiliations:** ^1^ Departamento de Biodiversidade, Evolução e Meio Ambiente Universidade Federal de Ouro Preto Ouro Preto Brazil

**Keywords:** chromosome evolution, karyotype, leafcutting ants, phylogenetic comparative methods, phylogenetic inference

## Abstract

Trait evolution has become a central focus in evolutionary biology, with phylogenetic comparative methods offering a framework to study how and why traits vary among species. Identifying variations in trait evolution rates within phylogenies is important for uncovering the mechanisms behind these differences. Karyotype variation, which is substantial across eukaryotic organisms, plays an essential role in species diversification. This study investigates karyotype variation within the leafcutting ant clade, focusing on chromosome number and morphology. We aim to determine whether karyotypic traits are phylogenetically dependent and how different evolutionary models explain karyotype diversity. Previous models have been insufficient in explaining these variations. To address these gaps, we employ modern phylogenetic methods to assess the impact of chromosomal fissions and fusions on karyotype evolution. By evaluating various evolutionary models—particularly the Brownian motion model, which suggests neutral chromosomal changes—we pursue for the further understanding the mode and tempo of karyotype evolution in ants. Our research examines how shifts in chromosomal change rates contribute to divergence among leafcutting ant species and assesses the role of chromosomal changes in the clade's evolutionary trajectory. Comparative analysis of leafcutting ant ideograms suggests that shared karyotype traits are strongly related to species relationships. This implies that karyotype diversification in leafcutting ants follows a phylogenetic trajectory at varying rates, with differences in karyotype traits reflecting the evolutionary distance between lineages. Particularly, the increase in the chromosome number of *Acromyrmex* is likely due to fission rearrangements rather than demi or polyploidization. We discuss and provide insights into the mechanisms driving karyotype variation and its implications for leafcutting ant diversification.

## Graphical abstract

Modern phylogenetic methods were used to explore how chromosome number and other karyotypic traits vary across species. The focus was on testing whether karyotypic traits follow a Brownian motion model (indicating neutral changes) or are influenced by phylogenetic constraints.This research elucidates the mechanisms underlying chromosomal evolution and its impact on leafcutting ant diversification.

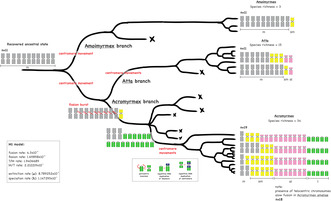

## 1 Introduction

The genome of any eukaryotic organism is sorted into protein‐upholder pieces called chromosomes that are morphologically distinguishable and specifically numbered in different taxa. The karyotype refers to the number and morphologies of the chromosomes, and are by the end the genome phenotype as their ultimate organizational architecture. Genes are distributed along the chromosomes in a non‐random way, prompting the cellular functions and then subject of the evolutionary processes. For instance, restriction to gene flow is a central trend in species diversification, and intrinsic barriers that impair gene flow can arrive through chromosomal mutation. Yet, there is still a debate whether chromosomal change is the cause or consequence of speciation (King 1993; Faria and Navarro 2010). Both can be the case, but certainly chromosomal changes have a huge impact on the insertion, deletion, or translocation of DNA sequences that can promote genome differentiation.

Karyotypes are particularly diverse in various animal groups but more or less uniform in the others, through distinct taxonomic levels. For instance, taking the fungus‐farming ants clade (ants that engage in a mutualistic relationship with basidiomycete fungi), all species from the *Acromyrmex* genus bear diploid karyotypes with 38 chromosomes, whereas *Atta* and *Amoimyrmex* have 22 chromosomes (Cardoso and Cristiano 2021). Yet the karyotypes known of the *Mycetophylax* genus vary from 26 to 36 chromosomes (Cardoso et al. 2014; Micolino et al. 2019; Micolino et al. 2022), whereas the known amplitude within the fungus‐farming clade itself ranges from 8 to 54 diploid chromosomes (Cardoso and Cristiano 2021). The asymmetry of the karyotype number and species richness across closely related groups is a conspicuous phenomenon in Hymenoptera (see Travenzoli et al. 2019) and still poorly understood. Although it is clear based on the growing evidence that chromosomal mutations may contribute to species differentiation (Rieseberg 2001), the understanding of the mechanisms and the direction and magnitude of karyotype conservation among closely related taxa is limited.

Since the earlier studies on ant chromosomes, the huge karyotypic diversity has aroused attention, and several models attempting to explain the processes governing chromosome number evolution in ants have been discussed. Three general hypothetic models of evolution state that the karyotype bears a starting chromosome number that can either increase or decrease (fission or fusion hypothesis, respectively) or diverge in both directions from the initial number of chromosomes (modal hypothesis) (Imai, Crozier, and Taylor 1977). All of them, according to Imai et al. (1986), fail to explain the karyotype variety of ants in some way, mainly due to DNA imbalance. Later, Imai et al. (1988) worked out the “minimum interaction hypothesis” that advanced based on empiric data from *Myrmecia* ants to “minimum interaction theory” (Imai, Taylor, and Crozier 1994). The theory states that rearrangements involving Robertsonian fissions are important, and karyotype usually tends toward an increase in the chromosome number and a decrease in the longitudinal chromosome size, seemingly helping to reduce the risk of deleterious rearrangements resulting from interactions between chromosomes in the nucleus (see Lorite and Palomeque 2010 for a review).

Here, we take advantage of modern phylogenetic correlative methods to analyze the chromosome number conservation in the clade of leafcutting ants. We are first interested in testing whether the chromosome number and other karyotypic traits are phylogenetically dependent. Our goal is to evaluate how fissions and fusions modulate the karyotype evolution of leafcutting ants. We evaluate the effectiveness of various evolutionary models in explaining the current interspecific variation in karyotypes and their features: the chromosome number and morphology and the number of chromosome arms. Our assumption is that if karyotypic traits can be explained by a Brownian motion (BM) model, it indicates that chromosomal changes occur in a more “neutral” manner, gradually over time. If they cannot, we need to explore how these changes relate to distinct phylogenetic transformations or if karyotypes evolve toward an adaptive optimum. How can shifts in the rate of chromosomal changes explain differences in divergence across leafcutting ant karyotypes? By evaluating shifts associated with chromosomal changes under phylogenetic constraints and recovering the ancestral character states, we can assess the mode and tempo of chromosomal evolution in the clade of leafcutting ants.

## 2 Materials and Methods

### 2.1 Phylogenetic Analysis

The phylogenetic hypothesis used to evaluate the dynamics of chromosomal change and best‐fitting evolutionary models were estimated through Bayesian inference (BI). We compiled nucleotide sequences from nuclear and mitochondrial genomic markers deposited in GenBank (Table S1) and merged with the alignment from Cristiano et al. (2020). Finally, five nuclear and two mitochondrial genomic fragments with 5596 base pairs of 161 operational taxonomic units (OTUs) in total were merged into a new sequence matrix (see Table S1). The sequences were aligned and concatenated using MEGA7 (Kumar, Stecher, and Tamura 2016). The ambiguously aligned sites were excluded (i.e., intronic region of *long‐wavelength rhodopsin* and mitochondrial tRNA‐Leucine), and the alignment was confirmed by translation to amino acids, whereas the missing data were coded as “?”. We cover most species, and the present study represents an extensive effort that vastly increased the number of leafcutting ant OTUs.

The phylogenetic inference was carried out by using Bayesian methods with Markov Chain Monte Carlo (MCMC). The selection of the most suitable model of molecular evolution that fits best to each potential partition was estimated by Partition‐Finder2 (Lanfear et al. 2012; Lanfear et al. 2017). We used Akaike's Information Criterion (AIC) and Bayesian Information Criterion (BIC) to select the best fitting models. Considering the estimated parameters (Table S2), we carried out a Bayesian analysis in MRBAYES v3.2.6 (Ronquist and Huelsenbeck 2003), which consisted of two independent runs of 20 million generations each, sampled every 1000 generations, and the convergence between runs was determined using TRACER v1.7.1 (Rambaut 2018). To i`nfer a calibrated hypothesis, we carried out a new BI inference using BEAST 2.5.2 (Bouckaert et al. 2014) under the fossilized birth–death (FBD) process (Heath, Huelsenbeck, and Stadler 2014) set to an uncorrelated log normal relaxed clock model (Drummond et al. 2006). We incorporated the occurrence times of fossil lineages (see Barden 2017) into the phylogenetic tree to impose a time structure and calibrate the analysis to absolute time. Fossil taxa were included as terminal nodes in the analyses and constrained to their corresponding monophyletic groups. The above‐mentioned nucleotide substitution scheme was used, and MCMC chains were run in two independent analyses with 100 million generations each, with sampling every 1000 generations. Convergence, mixing, and effective sample sizes (ESS > 200) were checked using Tracer v1.7.1. Sampled trees in each run were combined into a single file using LogCombiner 2.5.2, removing the first 10% of the trees in each run as burn‐in. A maximum clade credibility tree was generated by TreeAnnotator 2.5.2. Fossils used to calibrate the analyses were removed from the tree using the FullToExtantTreeConverter tool (as implemented in BEAUti 2.5.2). The generated tree and credible intervals were visualized in FigTree 1.4.3 (Rambaut 2009). The current version of ChromEvol can handle missing chromosome data; however, for the further analysis implemented in our study, taxa for which there is no chromosome data have been removed from the trees using the available pruning pipeline by Afonso Neto et al. (2022).

### 2.2 Cytogenetic Data Assembly

Cytogenetic data were assembled from the Ant Chromosome database available at www.ants.ufop.br (Cardoso, Santos, and Cristiano 2018 ). We further search in Web of Science and Scopus articles using the terms “fungus‐growing ants,” “Formicidae,” “chromosome,” and “karyotype”. The available data and respective references are shown in Table 1. We retrieve the available karyotypic formulae as published by the authors. Karyotypes determined by average chromosome measurements were considered the baseline of our study and were preferentially used in our analysis. The nomenclatures established by Levan, Fredga, and Sandberg (1964) based on the arms ratio (r) were consistently used here; thus, acrocentric (a) chromosomes are referred to as telocentric (t).

**TABLE 1:** Cytogenetic data of leafcutting ants assembled in this study.

Taxon	Haploid (*n*)	Diploid (2n)	Country/Localitty	Karyotyp e (2n)	Genome size Mpb (pg)	Traits					
						nm	nsm	nst	nt	fn	References
Leafcutting ants											
*Amoimyrmex striatus*	11	22	Brazil	20 m + 2sm	342.3 (0.35)	10	1	0	0	44	Cristiano, Cardoso, and Fernandes‐Salomão (2013) and Pereira et al. (2018)
*Amoimyrmex bruchi*	11	22	Argentina	20 m + 2sm	298.8 (0.31)*	10	1	0	0	44	Micolino et al. (2022)
*Amoimyrmex silvestrii*	11	22	Argentina	20 m + 2sm	311.37 (0.32)*	10	1	0	0	44	Micolino et al. (2022)
*Atta bisphaerica*	11	22	Brazil	12 m + 6sm + 4st		6	3	2	0	40	Fadini and Pompolo (1996)
*Atta colombica*	11	22	Panama	12 m + 6sm + 4st	298.8 (0.31)	6	3	2	0	40	Murakami, Fujiwara, and Yoshida (1998)
*Atta laevigata*	11	22	Brazil	12 m + 6sm + 4st	322.74 (0.33)	6	3	2	0	40	Fadini and Pompolo (1996)
*Atta sexdens*	11	22	Brazil	12 m + 6sm + 4st	322.74 (0.33)	6	3	2	0	40	Fadini and Pompolo (1996) and Santos‐Colares et al. (1997)
*Atta robusta*	11	22	Brazil	18 m + 2sm + 2st	332.52 (0.34)	9	1	1	0	44	Barros et al. (2015)
*Atta sexdens*	11	22	French Guiana	18 m + 2sm + 2st	322.74 (0.33)	9	1	1	0	44	de Aguiar et al. (2020)
*Acromyrmex ambiguus*	19	38	Uruguay	2 m + 6sm + 16st + 14 t	322.74 (0.33)	1	3	8	7	62	Goni et al. (1983)
*Acromyrmex ambiguus*	19	38	Brazil	14 m + 12sm + 8st + 4 t		7	6	4	2	68	Castro et al. (2020)
*Acromyrmex crassispinus*	19	38	Brazil	2 m + 6sm + 16st + 14 t	332.52 (0.34)	1	3	8	7	62	Fadini and Pompolo (1996)
*Acromyrmex crassispinus*	19	38	Brazil	12 m + 20sm + 4st + 2 t	332.52 (0.34)	6	10	2	1	74	Castro et al. (2020)
*Acromyrmex heyeri*	19	38	Uruguay	2 m + 6sm + 16st + 14 t		1	3	8	7	62	Goni et al. (1983) and Santos‐Colares et al. (1997)
*Acromyrmex lundi*	19	38	Brazil	10 m + 14sm + 10st + 4 t		5	7	5	2	72	Castro et al. (2020)
*Acromyrmex nigrosetosus*	19	38	Brazil	12 m + 14sm + 10st + 2 t	342.3 (0.35)	6	7	5	1	74	Castro et al. (2020)
*Acromyrmex hispidus*	19	38	Uruguay	2 m + 6sm + 16st + 14 t		1	3	8	7	62	Goni et al. (1983)
*Acromyrmex molestans*	19	38	Brazil	2 m + 6sm + 16st + 14 t	332.52 (0.34)	1	3	8	7	62	Fadini and Pompolo (1996)
*Acromyrmex molestans*	19	38	Brazil	10 m + 10sm + 16st + 2 t	332.52 (0.34)	5	5	8	1	74	Teixeira et al. (2017)
*Acromyrmex subterraneus*	19	38	Brazil	2 m + 6sm + 16st + 14 t	342.3 (0.35)	1	3	8	7	62	Fadini and Pompolo (1996)
*Acromyrmex subterraneus*	19	38	Brazil	14 m + 18sm + 4st + 2 t	342.3 (0.35)	7	9	2	1	74	Castro et al. (2020)
*Acromyrmex balzani*	19	38	Brazil, French Guiana	12 m + 10sm + 14st + 2 t	361.86 (0.37)	6	5	7	1	74	Barros et al. (2016), de Aguiar et al. (2020)
*Acromyrmex coronatus*	19	38	Brazil	12 m + 8sm + 16st + 2 t	332.52 (0.34)	6	4	8	1	74	Barros et al. (2016)
*Acromyrmex disciger*	19	38	Brazil	10 m + 12sm + 14st + 2 t	322.74 (0.33)	5	6	7	1	74	Barros et al. (2016)
*Acromyrmex echinatior*	19	38	Brazil	8 m + 6sm + 14st + 10 t	335 (0.36)	4	3	7	5	66	Barros et al. (2016)
*Acromyrmex niger*	19	38	Brazil	12 m + 14sm + 10st + 2 t	352.08 (0.36)	6	7	5	1	74	Barros et al. (2016)
*Acromyrmex rugosus*	19	38	Brazil	16 m + 12sm + 8st + 2 t	342.3 (0.35)	8	6	4	1	74	Barros et al. (2016)
*Acromyrmex aspersus*	19	38	Brazil	8 m + 10sm + 16st + 4 t		4	5	8	2	72	Teixeira et al. (2017)
*Acromyrmex ameliae*	18	36	Brazil	2 m + 8sm + 20st + 6 t		1	4	10	3	66	Barros et al. (2021)
*Acromyrmex brunneus*	19	38	Brazil	2 m + 6sm + 20st + 10 t	332.52 (0.34)	1	3	10	5	66	Barros et al. (2021)
Out groups											
Mycetomoellerius holmgreni	10	20	Brazil	20 m	322.74 (0.33)	10	0	0	0	40	Cardoso, Heinze et al. (2018)
Mycetomoellerius iheringi	10	20	Brazil	20 m	391.2 (0.40)	10	0	0	0	40	Micolino, Cristiano, and Cardoso (2020)
*Trachymyrmex septentrionalis*	10	20	Panama	20 m	244.5 (0.25)	10	0	0	0	40	Murakami, Fujiwara, and Yoshida (1998)

*Note:* Haploid (*n*) and diploid (2n) are given, followed by locality, karyotypic formula, and genome size available in Moura, Cardoso, and Cristiano (2021). The haploid karyotypes used in the present study are characterized by the number of metacentric, submetacentric, subtelocentric, and telocentric chromosomes, as well as the number of arms or fundamental number (fn). All nomenclature follows Levan, Fredga, and Sandberg (1964). *unpublished data.

### 2.3 Phylogenetic Signal, Rate Shifts, and Ancestral State Reconstruction of Karyotype Traits

Karyotype traits (chromosome number [*n*], fundamental number or chromosome arm number [fn], and the numbers of telocentric/acrocentric [t], subtelocentric [st], submetacentric [sm], and metacentric [m] chromosomes) were used as quantitative continuous characters (e.g., *n* is used as a continuous character; see Kandul, Lukhtanov, and Pierce 2007; Vershinina and Lukhtanov 2017). In fact, the models implemented in ChromEvol 2.0 consider the haploid chromosome number as an integer (Glick and Mayrose 2014; Rice and Mayrose 2020). We further used the function “transformPhylo.ML” in the packed MOTMOT (Puttick et al. 2020) in software R 3.2.1 (R Core Team 2022), which scales our karyotype traits by log‐transformation prior to the phylogenetic comparative analysis. The following packages have been used: *ape* (Paradis, Claude, and Strimmer 2004), *geiger* (Harmon et al. 2008), and *phytools* (Revell 2012).

First, we tested phylogenetic signals for all karyotype traits by calculating Moran's I (1948), Abouheif's *C*_mean_ (1999), Pagel's Lambda (1999), and Blomberg, Garland Jr, and Ives (2003). Then, we used these traits under AIC corrected for small sample size to test the best‐fitted tree transformation model of evolution by means of function “transformPhylo.ML” within the MOTMOT package in the R environment. Thus, we could evaluate the modes of karyotype evolution through the natural history of leafcutting ants. First, we fitted our data to a simple BM model that describes a process in which traits are modeled under the assumption of multivariate normal distribution (parameter σ^2^), meaning the null hypothesis of phylogenetic trait evolution (Felsenstein 1985). We then fitted our data to additional models that incorporate extra parameters and phylogenetic tree transformations to compare with the one‐rate BM model. These models include additional parameters: lambda (λ) accounts for the extent to which trait variation covaries with phylogenetic distance, allowing for the incorporation of variable evolutionary rates across different branches of the phylogenetic tree; delta (δ) parameter models how the evolutionary rate changes over time, capturing patterns of acceleration or deceleration in trait evolution; kappa (κ) parameter indicates that evolutionary changes occur at speciation events but are not necessarily proportional to branch length, allowing for variability in the rate of trait evolution across the phylogenetic tree; Ornstein–Uhlenbeck (OU) parameter (α) traits undergo a random walk within a constrained trait space, with a tendency to stabilize around an optimal value (Harmon 2019). The model that fits best in explaining karyotype trait evolution was evaluated under Akaike Information Criterion corrected to small sampling size (AICc). We also test for rate shifts of karyotype trait evolution across the phylogeny by using *traitMEDUSA* (for details, see Puttick, Thomas, and Benton 2014).

Shifts in evolutionary rates were estimated by applying the *traitMEDUSA* function in *motmot*. To detect the rate shifts, *traitMEDUSA* fits the data to a simple one‐rate BM model, in which the likelihood will be compared under Akaike Information Criterion corrected to small sampling size (AICc) with the likelihood of two‐rate models at each node across the phylogeny. We used this method to recover rate shifts occurring throughout the leafcutting ant clade without specifying any particular node a priori. We use a default and conservative cut‐offs of four and nine values, respectively (Thomas and Freckleton 2012). As mentioned before, we used the function “transformPhylo.ML” with the tm2 algorithm to set the *traitMEDUSA* model.

We further use ChromEvol v2.0 (Glick and Mayrose 2014) to model chromosome evolution under a statistical framework according to parameters such as gain or loss of a single chromosome (dysploidy), polyploidy or demi‐polyploidy, and the dependency between the current haploid number and the rate of gain and loss of a chromosome (constant or linear), estimating alongside the ancestral node number.

**FIGURE 1:**
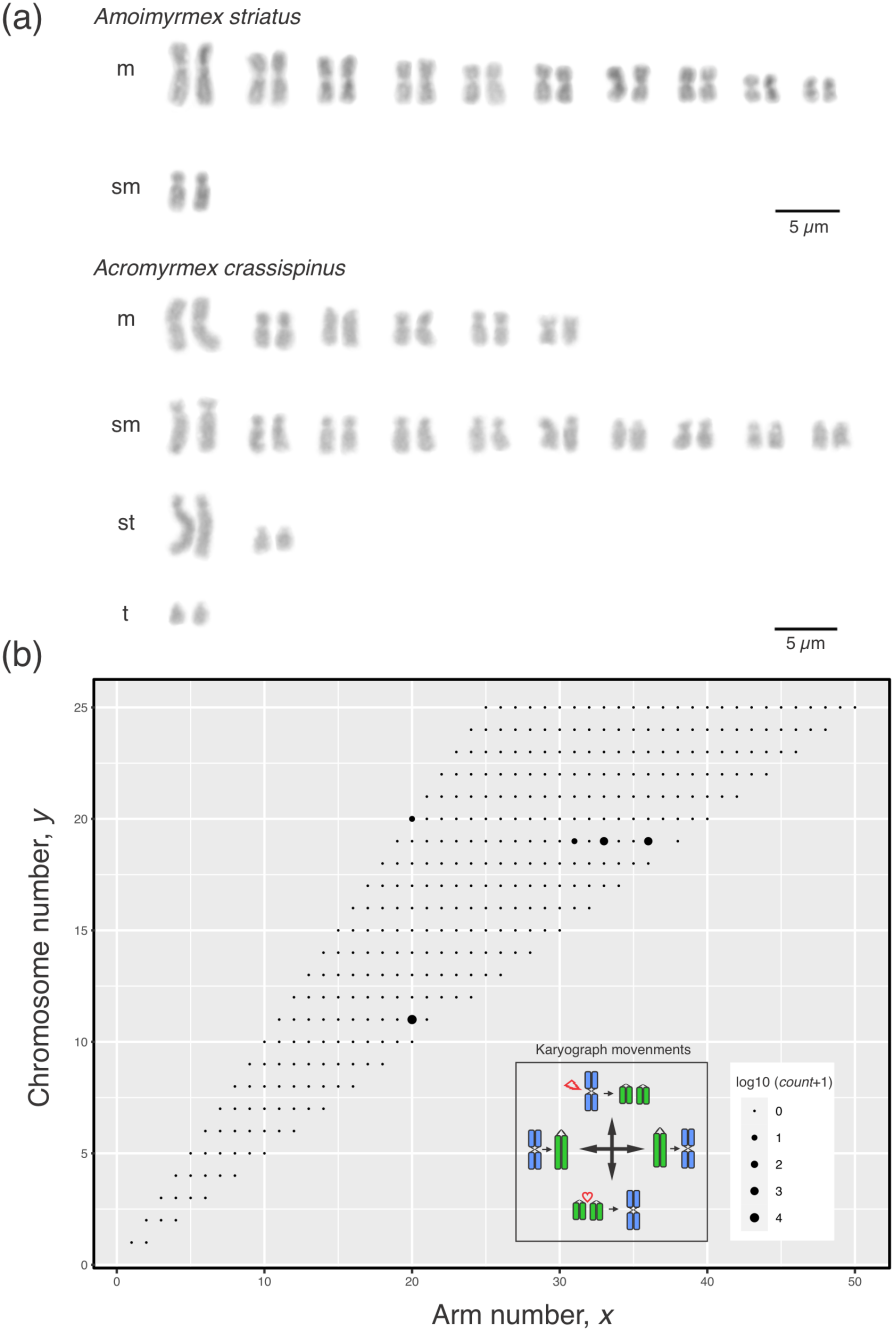
Karyotypes and karyograph of leafcutting ants. (a) Representative karyotypes of leafcutting ants depicting the 11 pairs (*n* = 11) of metacentric (m) and submetacentric (sm) chromosomes of *Amoimyrmex striatus* and the 19 pairs (*n* = 19) of metacentric (m), submetacentric (sm), subtelocentric (st), and telocentric (t) chromosomes of *Acromyrmex crassispinus*. (b) The karyograph of leafcutting ants estimated considering the data from Table 1. Movements in the karyograph bidimensional space are represented in the inset showing fusion and fissions in the *y*‐axis and the transition from the m to t chromosome and vice versa in the *x*‐axis.

Finally, we independently modeled leafcutting ant chromosome evolution by using a probabilistic method that simultaneously incorporates the chromosome number and morphology under the bidimensional scale of a karyograph (Figure 1). The model estimates the ancestral number of chromosomes plus four/five parameters (k1–5) in a MUSSE analysis under maximum likelihood estimation incorporating speciation (λ) and extinction rates (μ). The parameter k1 is the fusion rate coefficient, k2 is the fission rate coefficient, k3 is the telocentric‐metacentric transition rate coefficient, whereas k4 is the metacentric‐telocentric transition rate coefficient, yet the parameter k5 includes the polyploidization rate into the model (Yoshida and Kitano 2021). We run two different models: model 1 (M1) without the polyploidization parameter and model 2 (M2) including k5, or polyploidization parameter. The fit of the models was calculated by using the likelihood ratio test (LTR).

## 3 Results

### 3.1 Phylogeny Reconstruction, Karyotype Traits, and Phylogenetic Signals

**FIGURE 2:**
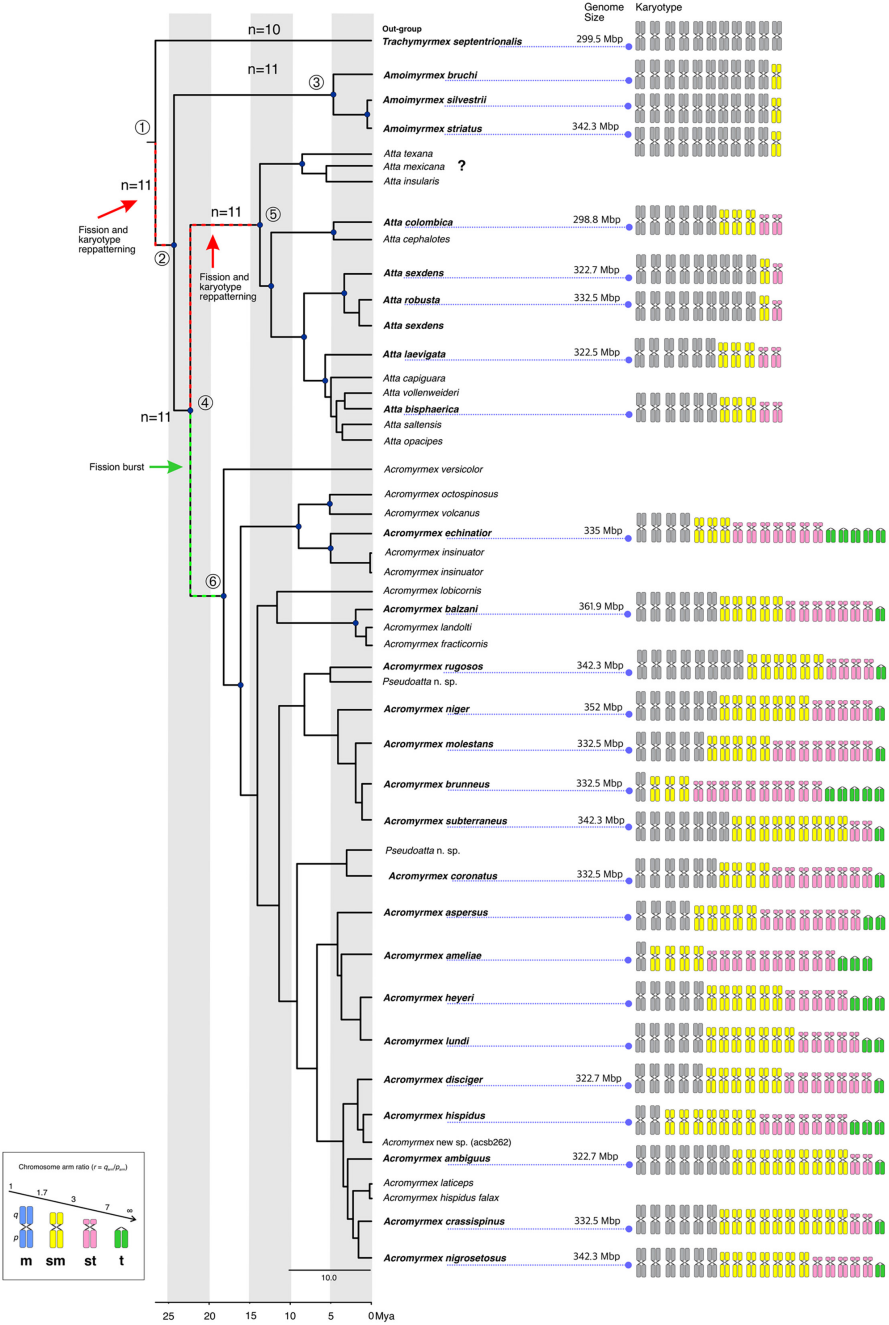
Phylogenetic relationships of leafcutting ants and ideograms presenting the karyotype traits based on Levan, Fredga, and Sandberg (1964) arms ratio from the data compiled in Table 1. Haploid chromosome number (*n*), chromosome morphologies (m, sm, st, and t), and fundamental number (fn) are given. Additionally, genome size estimates are depicted when available (from Moura, Cardoso, and Cristiano 2021). The blue dots at nodes indicate the posterior probability above 0.95. The numbers depict the main clades: 1: Higher Attina, 2: Leafcutting ants, 3: *Amoimyrmex* clade, 4: *Atta*+*Acromyrmex* clade, 5: *Atta* clade, and 6: *Acromyrmex* clade.

The phylogenetic relationships revealed through BI are similar to previous molecular phylogenetic studies (Cristiano, Cardoso, and Fernandes‐Salomão 2013; Cristiano et al. 2020) comprising leafcutting ants. We found strong support for monophyly of the leafcutting ants and their genera (Figure 2), the central taxonomic level of our study. *Amoimyrmex* includes the three known species and is considered a sister group of the clade that contains *Acromymex* and *Atta* species. This result is expected and consistent with previous findings also based on genomic data (e.g., Branstetter et al. 2017), but the results of our multilocus study are based on significantly expanded taxonomic coverage of the leafcutting ants. We found low resolution and weak support (posterior probabilities < 0.95) within the *Acromyrmex* clade, but we can clearly detect related species from Central America, including “*octospinosus*+*echinatior*”, a diverging “*lobicornis*+*balzani”* group, “*rugosos”* diverging from the “*niger”* group, and “*coronatus”* (only arboreal species) as a sister to the “*ambiguus”* and “*disciger”* groups. This is still to be confirmed by including more loci or genomic data. Further, the position of *Acromyrmex versicolor* as a sister group of remaining congeners is consistent with the previously reported results (Solomon et al. 2019).

Mapping the karyotype traits onto the leafcutting ant clade (Figure 2), we found that each genus is characterized by a specific morphological set of chromosomes. *Amoimyrmex* species with *n* = 11 exhibited only metacentric and submetacentric chromosomes, whereas *Atta* species with *n* = 11 includes metacentric, submetacentric and subtelocentric chromosomes. Yet, *Acromyrmex* species with *n* = 38 or 36 (in the parasitic species *Ac. ameliae*) were the only to exhibit telocentric beyond the metacentric, submetacentric, and subtelocentric chromosomes (Figure 2). We can suggest that the general karyological formula of *Amoimyrmex* would be *K* = “*n*” *m* + “*n*” sm; *Atta* would be *K* = “*n*” *m* + “*n*” sm + *“n*” st, whereas *Acromyrmex K* = “*n*” *m* + “*n*” sm + “*n*” st + “*n*” *t*, where “*n*” is the specific number of each chromosome.

When we tested the phylogenetic signal of the karyotype traits, we found that all traits are strongly phylogenetically correlated (Table 2), with the exception of nonsignificant Blomberg's K for the number of metacentric (nm) and telocentric (nt) chromosomes. However, Pagel's λ indicated a significant phylogenetic signal for all traits, with values from ~0.5 to 1 and significant *p—*values (Table 2), indicating that karyotype traits in leafcutting ants are phylogenetically correlated. All other autocorrelation estimators, Abouheif's *C*_mean_ and Moran's *I*, also indicate a phylogenetic dependence of karyotype traits (Table 2); thus closely related species are more similar than expected by chance.

**TABLE 2:** Phylogenetic signal in karyotype traits among leafcutting ants.

	Moran's *I*		Abouheif's *C*_mean_		Bloomberg's K		Pagel's λ	
	*I*	*P* (permutation)	*C*_mean_	*P* (permutation)	*K*	*P* (randomization)	λ	*P* (LTR test)
Chromosome number (*n*)	0.84	0.00	0.86	0.001	7.16	0.001	1.00	4.31E‐20
Number of metacentrics (nm)	0.29	0.01	0.31	0.011	0.28	0.104	0.41	0.024
Number of submetacentrics (nsm)	0.45	0.00	0.46	0.001	0.34	0.030	0.46	0.002
Number of subtelocentrics (nst)	0.42	0.00	0.44	0.001	0.32	0.050	0.50	0.003
Number of telocentrics (nt)	0.19	0.06	0.24	0.034	0.25	0.257	0.48	0.039
Fundamental number (fn)	0.81	0.00	0.82	0.001	2.32	0.001	0.93	9.05E‐11

### 3.2 Karyotype Trait Evolution, Rates, and State Reconstructions

**FIGURE 3:**
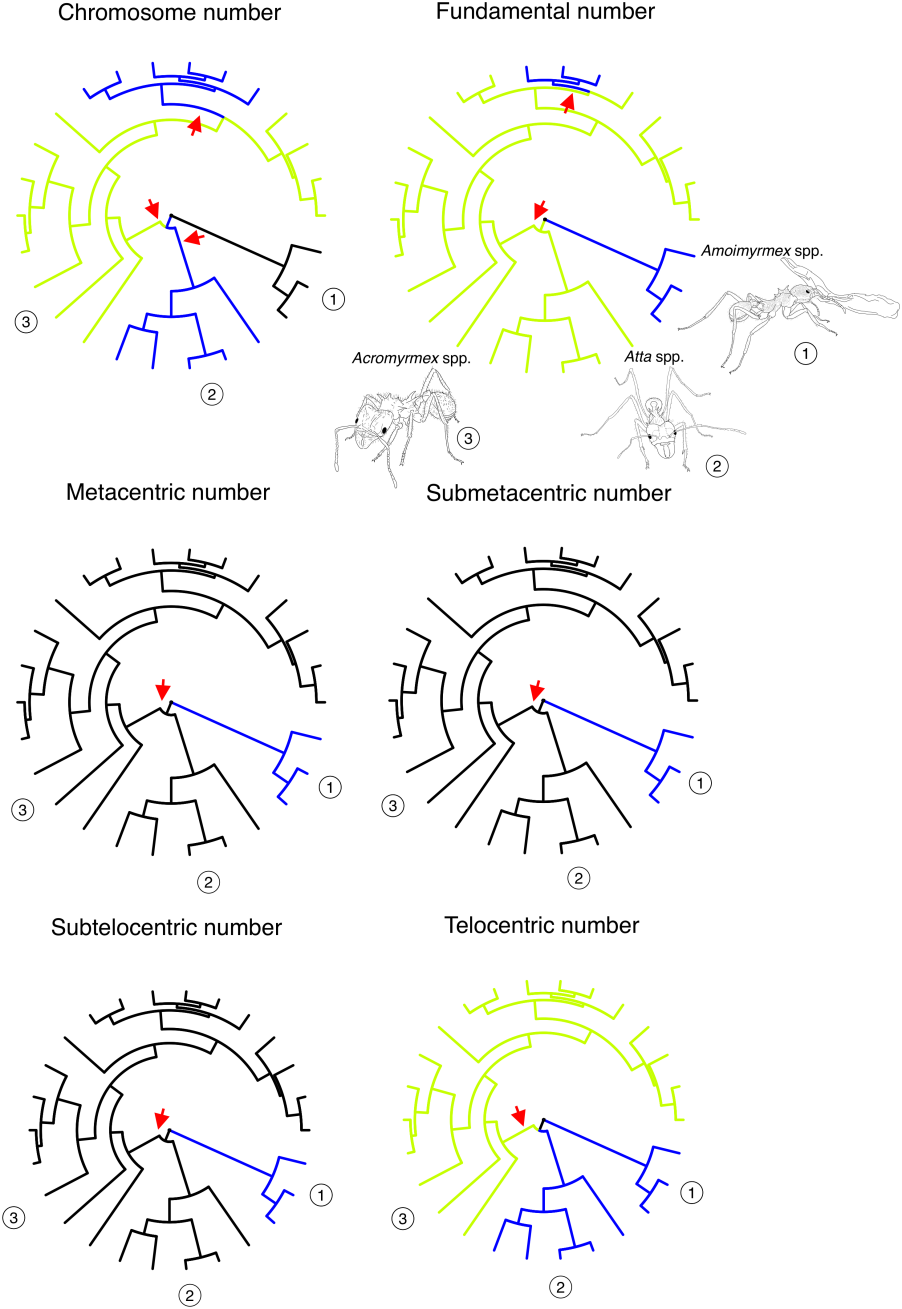
Evolutionary rate of karyotype changes, assuming the time‐calibrated tree of leaf‐cutting ants. Detected shifts for the karyotype traits as inferred throughout the phylogeny are indicated by colors. The red arrows show the stem branch of clades with support for whole clade shifts in evolutionary rate.

Here, we tested different models for karyotype trait evolution (Table 3). First, we evaluate the fit of karyotype traits to the BM model, in which traits are modeled approximately as a random walk across the phylogeny by the influence of stochastic factors. Then BM fit was compared to the fit of four tree transformation models with additional parameters (i.e., Lambda, Delta, and Kappa) that are commonly used to identify departures from a simple stochastic event. The test of the four different models for the karyotype traits suggests that evolutionary trajectories in the karyotype traits (chromosome number, nm, nsm, sst, and nt) were best explained by the Lambda model (Table 3), suggesting that the divergence of such karyotype traits covaries with the phylogenetic distance but allows for variable evolutionary rates. Yet, the BM model and the Lambda model explain the fundamental number (fn), indicating that the divergence in the number of chromosome arms in leafcutting ants is perfectly predicted by the phylogenetic distances, but the AICc of the Lambda model was slightly lower than that of the BM model, indicating it as the best‐fitting model (Table 3). To test shifts in the evolutionary rate of leafcutting ant karyotypes, we estimated the phylogenetic position of changes using trait MEDUSA (Figure 3). Clade‐based shifts in the evolutionary rate were identified by the models with three distinct rate shifts for the chromosome number (AICc = −165.967), two shifts for the fundamental number (AICc = 68.09727), and one shift for the remaining traits: metacentric number (AICc = 87.59428), submetacentric number (AICc = 83.56541), subtelocentric number (AICc = 94.06694), and telocentric number (AICc = −60.72876).

**TABLE 3:** Karyotype traits of leafcutting ants under different evolutionary scenarios.

Evolutionary models	Parameters	Chromosome number (*n*)	Number of metacentric (nm)	Number of submetacentric (nsm)	Number of subtelocentric (nst)	Number of telocentric (nt)	Fundamental number (fn)
Brownian motion	σ^2^	0.137	1.454	1.273	1.876	0.465	6.028
	Likelihood	36.610	67.295	65.564	70.604	52.484	85.779
	AIC	77.220	138.590	135.127	145.209	108.968	175.558
	AICc	77.741	139.112	135.649	145.731	109.490	176.080
	ΔAICc	19.401	15.5198	18.1335	15.4766	14.91172	0.3255
Lambda	λ	1.00000	0.4053	0.4628346	0.50491	0.47835	0.9268337
	Variance	0.1372586	0.2623182	0.2168264	0.3664729	0.09087273	4.029888
	AIC	79.21973	122.50130	116.42460	129.16350	93.48837	174.66260
	AICc	80.31063	**123.59220**	**117.51550**	**130.25440**	**94.57928**	**175.75350**
	ΔAICc	21.970	0.00*	0.00*	0.00*	0.00*	0.00*
Delta	δ	0.056	5.000	5.000	5.000	5.000	0.703
	Variance	13.664	1.14E‐06	0.000	0.000	0.000	19.94
	AIC	57.249	130.078	126.363	138.868	101.998	177.193
	AICc	**58.340**	131.169	127.454	139.959	103.088	178.284
	ΔAICc	0.00*	7.5768	9.9385	9.7046	9.7046	2.5305
Kappa	κ	1.00000	0.00	0.00	0.00	0.12	0.52
	Variance	0.137	2.681	1.92	3.25	0.85	9.70
	AIC	79.21973	127.72	119.05	132.69	101.02	175.42
	AICc	80.31063	128.81	120.14	133.78	102.11	176.51
	ΔAICc	21.970	5.2178	2.6245	3.5256	3.5256	0.7505
OU	**α**	0.000	2.00	0.25	2.00	2.00	0.00
	Variance	0.137	26.18	3.08	39.38	8.30	54.24
	AIC	79.220	127.63	123.43	138.24	97.77	177.56
	AICc	80.310	141.68	138.22	148.30	112.06	178.51
	ΔAICc	21.970	18.0878	20.7045	18.0456	18.0456	2.7565

*Note:* Best‐supported models of the relationship of each karyotype trait are shown in bold. For each trait, the model with the lowest Akaike information criterion corrected for small sample size (AICc) scores the best‐fitting model and ΔAICc. Model parameters: σ^2^, net rate of trait evolution in the Brownian motion model; λ, extent to which phylogeny predicts covariance among trait for species; δ, comparing the contribution of early versus late trait evolution across a phylogeny; κ, evolutionary change in trait associated with speciation events along the branch length; α, evolutionary constraint parameter in the Ornstein–Uhlenbeck (OU) model moving trait values back to the optimum. *best‐fitting model.

We also tested rates of chromosomal changes by modeling the number of chromosomes and number of arms under the karyograph space (see Figure 1b), considering extinction and speciation rates across leafcutting ant phylogeny. The log‐likelihood of the M1 model was −342.2464, and the coefficient rates estimated were as follows: k1 = 4.3 × 10^−9^, k2 = 1.69898 × 10^−2^, k3 = 1.940689, k4 = 2.212219 × 10^−1^, λ = 1.247293 × 10^−1^, and μ = 8.789252 × 10^−2^, whereas the log‐likelihood of M2 was −331.4004, and the coefficient rates were as follows: k1 = 1.2663434 × 10^−2^, k2 = 4.495113 × 10^−3^, k3 = 1.544296962, k4 = 1.92182173 × 10^−1^, k5 = 9.674833 × 10^−3^, λ = 1.25029844 × 10^−1^, and μ = 8.8269229 × 10^−2^. The reconstructed evolutionary trajectories were overall similar between the M1 and M2 models, but the model that includes polyploidization (k5) was rejected. Being the M1 best‐fitting model to explain chromosome changes in leafcutting ants, the 2ΔLnL value was 21.692 and the *p‐*value was 0.99, accepting the null hypothesis.

**FIGURE 4:**
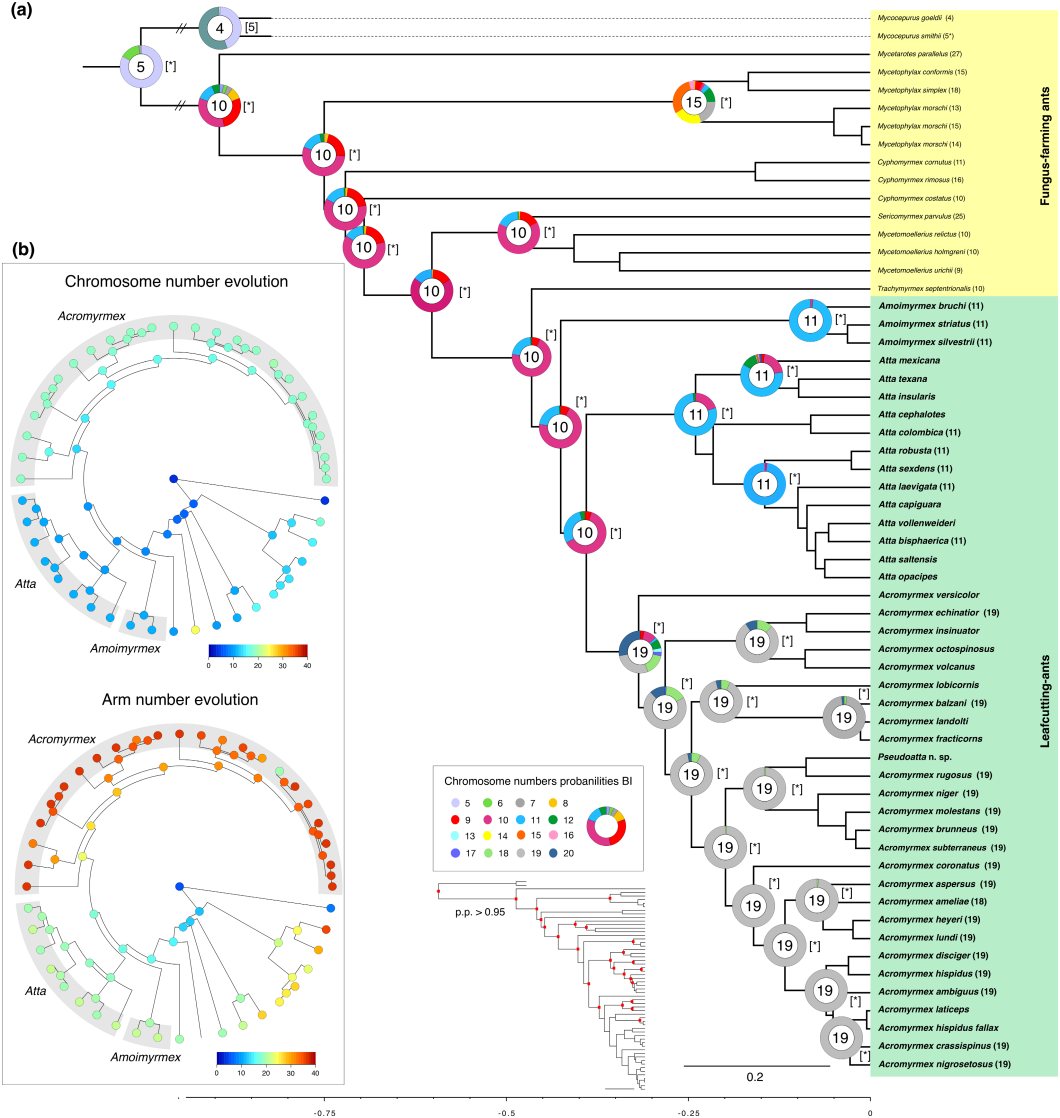
Ancestral haploid chromosome number as recovered by ChromEvol 2.0 under BI and ML inference and by model M1 under karyograph method. (a) The ancestral chromosome number with the highest probability is given inside the circle and pie charts at the main nodes. The colors on the pie charts represent the proportional probability of each given chromosome number according to the legend. The known karyotypes of species are given at the tip. The haploid ancestral chromosome numbers with the best likelihood are given in brackets. Asterisks represent the same estimated haploid number in BI. (b) The circles at the nodes indicate heat maps of mean values of chromosome numbers (a) and arm numbers (b) in the marginal ancestral reconstruction of karyotypes inferred with the M1 model under karyograph space. The colored points on the tips indicate the karyotype of extant species.

For the reconstruction of ancestral states of karyotype traits, we used two distinct methods, one modeled by ChromEvol 2.0 models and another based on karyograph (see Figure 1b) bidimensional space that takes into account the numbers of chromosomes and chromosome arms (or the fundamental number implying that all metacentric, submetacentric, and subtelocentric chromosomes have two arms, whereas telocentric have only one arm), as well as speciation and extinction rates along the phylogeny, as estimated in the M2 model. In both cases, fissions, fusions, and polyploidization are considered, and the ancestral states at nodes are estimated by using maximum likelihood and/or BI (Yoshida and Kitano 2021). The best‐fitting model that better explains the chromosome trajectories as estimated by ChromEvol2.0 implies that fissions, fusions, and demi‐duplication events better explain chromosome changes in leafcutting ants (AIC = 149.4). The most likely ancestral karyotype of the most recent common ancestor (MRCA) of leafcutting ants bears a haploid set of 10 (> P.P.) or 11 chromosomes (< P.P.), as well the MRCA of the clade *Atta* + *Acromyrmex* (Figure 4a). The recovered state for *Amoirmyrmex* and *Atta* MRCA was a haploid chromosome set of 11 (> P.P.), whereas 19 followed by 18 chromosomes in the haploid set showed the higher posterior probabilities for the *Acromyrmex* clade. All results inferred by BI were corroborated to the results obtained by maximum likelihood estimations. The models implemented under the karyograph constraint are very much congruent with the estimation of ChromEvol 2.0 (Figure 4b). Additionally, the karyograph constraint model recovered the ancestral states of arm numbers that suggest that fissions followed by duplications of chromosome segments or inversion events have driven karyotypic change in leafcutting ants since the MRCA (Figure 4b).

## 4 Discussion

Here, we explored for the first time the phylogenetic dependence of karyotypic traits (chromosome number and morphology) in an ant group. We found a strong phylogenetic signal in all evaluated karyotypic traits among leafcutting ants, despite the conservative nature of the chromosome number within genera. All studied species of the genera *Amoimyrmex* and *Atta* consistently exhibit 11 pairs of chromosomes, whereas *Acromyrmex* species typically have 19 chromosome pairs (reviewed in Cardoso and Cristiano 2021). While each genus demonstrates numerical stability in the chromosome number, there is notable variation in chromosome morphology that is consistent within genera. *Acromyrmex*, the most species‐rich genus, exhibits the greatest diversity in chromosome morphology types, featuring chromosomes with various centromere positions and exclusively telocentric chromosomes. In contrast, the karyotypes of *Atta* and *Amoimyrmex* exclusively consist of biarmed chromosomes. Based on these findings, it appears that closely related species of ants exhibit more similarities in their karyotype traits compared to species that are less closely related. This suggests that karyotype traits are phylogenetically correlated, supporting the idea proposed by Lorite and Palomeque in 2010. According to their hypothesis, changes in the karyotype have accompanied the differentiation of species within Formicidae. Phylogenetic correlations in the chromosome number have been observed across various taxonomic levels in distinct groups of animals and plants (Kandul, Lukhtanov, and Pierce 2007; Vershinina and Lukhtanov 2017; Carta, Bedini, and Peruzzi 2018; Márquez‐Corro et al. 2021).

The comparative analyses of karyotype trait variation within the leafcutting ant clade indicate that there is a noticeable phylogenetic pattern. This pattern suggests that the extent of karyotypic divergence among lineages is strongly influenced by the time since they diverged from a common ancestor; thus, the earlier the two lineages split from each other, the more distinct their karyotype traits tend to be. Such observation aligns with the concept of the molecular clock in evolutionary biology, where genetic and phenotypic differences accumulate gradually over time. Whereas the pattern is consistent with the molecular clock concept, it is not explained by a constant rate of trait evolution among lineages since a simple BM model of neutral evolution does not work well in this case.

In fact, we found that evolutionary diversification in karyotype traits of leafcutting ants was best supported by the Delta model, indicating that the evolutionary trajectory in the number of chromosomes (*n*) accelerated or decelerated within lineages. At first glance, this can be clearly observed in the *Amoimyrmex* clade or *Atta* clade versus *Acromyrmex* clade (Figure 2). Yet, chromosome morphologies (nm, nsm, nst, and nt) and the number of chromosome arms (fn) were best explained by the Lambda model, which suggests that clades and lineages of leafcutting ants are characterized by variable rates of karyotype trait evolution. We detected distinct rate shifts among clades of leafcutting ant phylogeny regarding all karyotype traits (see Figure 3). Taken together, our results suggest that karyotype evolution varies significantly among lineages, with some exhibiting rapid changes while others remain stable over time. This indicates that evolutionary rates for karyotype traits are not uniform but differ between lineages at different times. Thus, it appears that the contemporary chromosome number and morphology are influenced not only by neutral evolution and genetic drift but also by selection and other evolutionary constraints. For instance, constraints imposed by the size of the chromosomes, as postulated by the “minimum interaction theory” (Imai, Taylor, and Crozier 1994), and an expected limit in the number of chromosomes (see Cardoso and Cristiano 2021) may be reliable mechanisms influencing the evolution of leafcutting ant karyotypes.

Considering the fundamental number, the BM model was also suitable for explaining the evolution of chromosome arms, suggesting that such karyotypic traits can gradually increase and accumulate independently, unlike other traits evaluated here. However, reducing the karyotype to arm number obscures the complete karyotypic information, as metacentric, submetacentric, and subtelocentric chromosomes are all grouped together.

**FIGURE 5:**
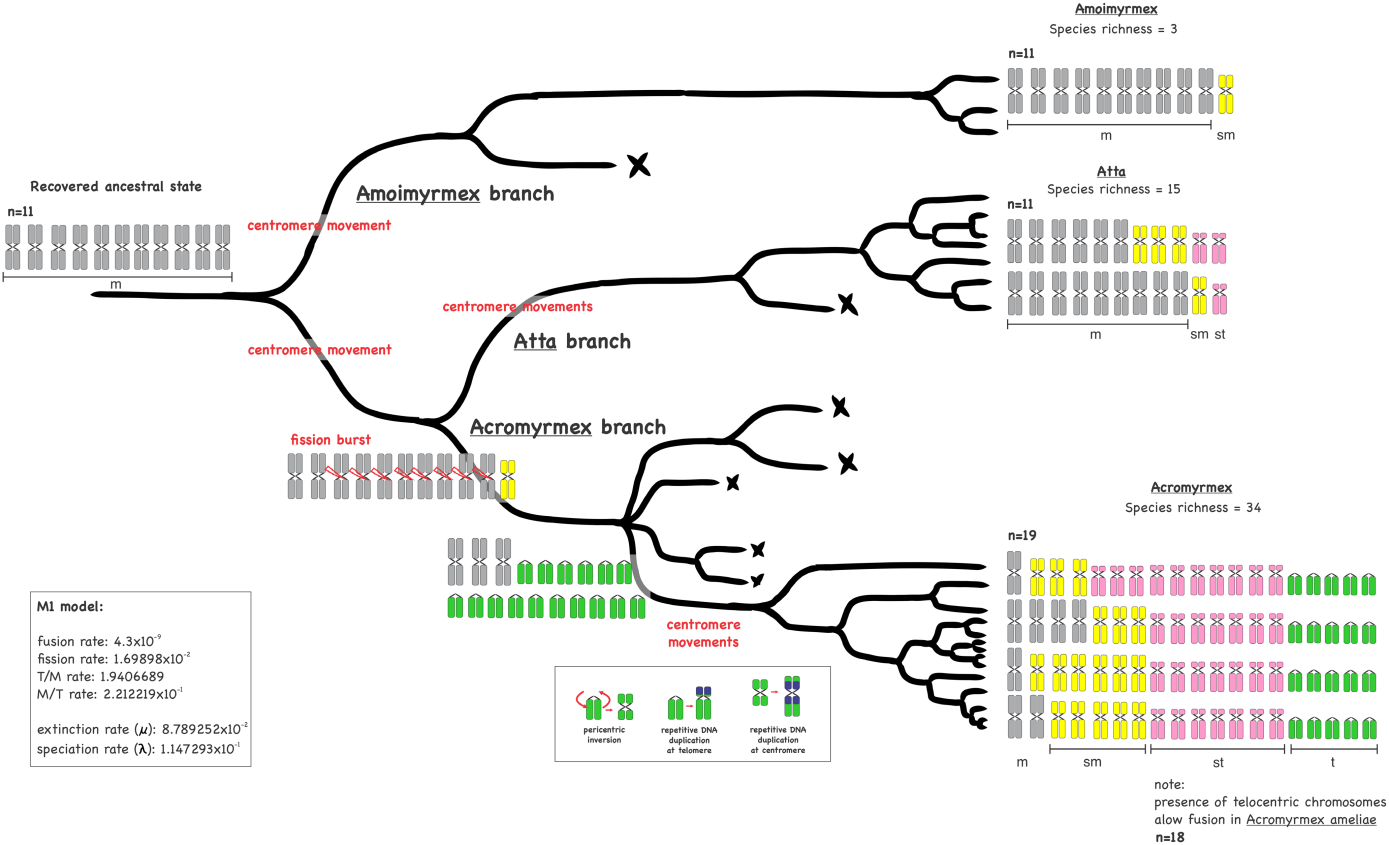
Schematic scenario for karyotype changes in leafcutting ants. Chromosome number evolution was inferred by ancestral chromosome state under Bayesian and Maximum Likelihood optimization using the M1 model of the karyograph method. The ancestral state was metacentric with *n* = 11 chromosomes. To the right of each clade of leaf‐cutting ants is depicted the general chromosome morphology comprising the karyotypes observed in known karyotypes. Likely rearrangements undergone during karyotype evolution are highlighted at each branch. Coefficient rates estimated by the model in the inset show that the fission rate and the movement from uniarmed (T/M) to biarmed (m, sm, and st) chromosomes were higher than the fusion and M/T coefficient rates. Speciation rates were higher than extinction rates. The shared number of chromosomes between *Atta* and *Amoimyrmex* indicates more stable arrangements in linkage groups of chromosomes. The lineage leading to *Acromyrmex*, which breaks such a karyotype structure, has a diversified set of existing karyotypes with *n* = 19. New fusion events can arise, as observed in *Acromyrmex ameliae* (*n* = 18), a parasitic ant species (see discussion for details). Species richness retrieved from www.antcat.org.

Our current study confirms the previously estimated putative chromosome number of the MRCA of leafcutting ants (Cristiano, Cardoso, and Fernandes‐Salomão 2013; Pereira et al. 2018). We estimated the haploid number as *n* = 11, followed by *n* = 10 chromosomes with the higher posterior probabilities (Figure 2). The ancestral reconstruction by ChromEvol implements a Bayesian approach that yields posterior probabilities around each recovered inference, providing a statistically supported parameter. Furthermore, all other methods similarly recovered this ancestral state, even considering the karyograph method that takes the chromosome morphology into account. We hypothesize that the ancestral karyotype was likely characterized by metacentric and submetacentric chromosomes, as this configuration best explains the chromosome changes of the leafcutting ants and the respective changes in karyotype traits. First, we need to mention that it is evident from our study that chromosomal rearrangements accompanied the diversification of both leafcutting ant genera. Second, the karyotyped species of *Amoimyrmex*, a sister group of the remaining leafcutting ants, present a conserved number of *n* = 11 of metacentric/submetacentric chromosomes. The karyotyped species of *Atta* clade also have *n* = 11 (Figure 2). Third, the sister group of leafcutting ants, *Trachymyrmex*, is recorded to be *n* = 10 with all metacentric chromosomes (see Table 1). Considering these arguments, we can undoubtedly define that the ancestor of leafcutting ants likely had *n* = 11/10 chromosomes, most of which were presumably metacentric (Figure 5).

Changes in the number of chromosomes are frequently observed in nature, and shifts in karyotypic traits can result from events such as chromosome fusion or fission, polyploidization, or translocation. Taking this as a starting point together with the well‐substantiated knowledge of karyotype evolution of ants (Imai et al. 1986, 1988; Imai, Crozier, and Taylor 1977; Imai, Taylor, and Crozier 1994; Imai, Satta, and Takahata 2001), there may be a trend for an increase in the chromosome number, mediated by an unbalanced fission–fusion ratio. Apparently, the initial diversification of leafcutting ants into three genera was not mediated by numerical chromosomal changes in any specific lineage, accompanied by any particular structural rearrangements at any specific rate. *Amoimyrmex* karyotypes bear only one submetacentric chromosome pair (Cristiano, Cardoso, and Fernandes‐Salomão 2013; Micolino et al. 2022), whereas *Atta* karyotypes can bear up to three submetacentric pairs plus subtelocentric pairs (Table 1). Thus, rearrangements such as deletions or inversions, likely mediated by repetitive DNA, must have contributed to the karyotypes observed in the present study. In fact, distinct classes of repetitive DNA, such as microsatellites (Micolino, Cristiano, and Cardoso 2019) and centromeric sequences (Carta, Bedini, and Peruzzi 2018), have been proposed to impair gene flow through karyotype differentiation. Furthermore, although rarely documented, a single inversion distinguishes the karyotype of sympatric species in *Mycetomoellerius* (Micolino, Cristiano, and Cardoso 2020), closely related to leafcutting ants. Thus, with a slow rate of chromosomal change in *Amoimyrmex* and a slightly faster rate in *Atta*, the centromere has been repositioned, resulting in the formation of submetacentric and subtelocentric chromosomes (Figure 5).

After the split between *Atta* and *Acromyrmex*, a significant shift in the rate of chromosomal change occurred in the lineage that diversified into *Acromyrmex* (Figures 2 and 5). The predominant chromosomal rearrangements during this diversification were fissions (the parameter k2—fission coefficient was three times higher than other parameters in the most likely model M2). Previous cytogenetic studies have proposed that chromosomal fissions played a key role in *Acromyrmex* karyotype evolution (Barros et al. 2016). These fissions were likely followed by other structural rearrangements, such as pericentric inversions and expansion (duplications) of repetitive DNA around telomeres and centromeres (Imai, Taylor, and Crozier 1994). Duplications of repetitive DNA have also accompanied the phylogenetic divergence of psammophilous *Mycetophylax* (Cardoso, Moura, and Cristiano 2022). These processes likely resulted in the contemporary karyotypes observed in *Acromyrmex*, composed of 19 pairs of chromosomes ranging from metacentric to telocentric (Figure 5).

Considering karyotype conservatism of *Atta* and *Amoimyrmex* and the rates of fission–fusion (k1 and k2) and centromere movement (k4 and k3) here, we propose that a fission burst rapidly changed the ancestral karyotype from *n* = 11/10 to *n* = 19, which then rapidly diversified into the numerous *Acromyrmex* species (see Figure 5). Chromosome‐level analysis in butterflies has concluded that fissions and fusions contribute to species divergence (Mackintosh et al. 2023). Likewise, small chromosomes are expected to show a high recombination rate, resulting in higher nucleotide diversity and sequence divergence (Tigano et al. 2022). Considering such an idea, along with the shift in the rate of chromosomal change in *Acromyrmex* (Figure 5), the species richness, and the chromosome morphology variation observed (see Figures 2 and 5), it is plausible to assume that the ancestral lineage experienced a major transition to the *n* = 19 karyotype, producing a unique new genome architecture that broke the ancestral organization. The ongoing repatterning through substructural rearrangements has produced the existing diversity, both in species and karyotype structure. Indeed, we recovered additional shifts in the rate of chromosome change within the *Acromyrmex* clade. Also, one fusion is suggested to have occurred in the distinct karyotype of the *Acromyrmex* that harbors a haploid chromosome set of 18 chromosomes (Barros et al. 2021).

Importantly, the transition from a lower number (*n* = 11/10) of metacentric chromosomes to a higher number (*n* = 19), predominantly composed of subtelocentric chromosomes in leafcutting ants, aligns with the “minimum interaction theory” (Imai, Taylor, and Crozier 1994), as well as with the concept of female meiotic drive (King 1993; White 1978). According to the former theory, the number of chromosomes in karyotypes increases through centric fissions, while chromosome size decreases. This process minimizes the risk of deleterious rearrangements caused by non‐homologous interactions of large chromosomes within the nucleus. The latter suggests that in heterokaryotypes, chromosomes with a specific morphology are preferentially transmitted to the egg rather than polar bodies, thus deviating from the random segregation. Thus, the rates at which chromosome morphologies change over time can provide clues for evaluating the role of such mechanisms in karyotype evolution. The fission–fusion bias (k2/k1) in our models aligns with the “minimum interaction theory”, while the transition bias (k4/k3), which involves the movement of centromeres, fits the concept of meiotic drive and centromere drive (see Cardoso et al. 2018).

Finally, while the models recovered the polyploidization rate, it seems unlikely that such events took place in the chromosome evolution of leafcutting ants. Some evidence argues against whole‐genome duplication in the karyotype evolution of leafcutting ants. Among these arguments are: the genome size observed in *Amoimyrmex* and *Atta* species compared to *Acromyrmex* species is all around 300 Mbp (Moura, Cardoso, and Cristiano 2021); the high number of subtelocentric and telocentric chromosomes (see Figure 2); and the proportion and localization of positive heterochromatic blocks revealed by C‐bands in the karyotype of *Amoimyrmex* and *Atta* versus *Acromyrmex* (Murakami, Fujiwara, and Yoshida 1998; Cristiano, Cardoso, and Fernandes‐Salomão 2013; Barros et al. 2014, 2016; Moura, Cardoso, and Cristiano 2021). The “minimum interaction theory” predicts beyond the unbalanced fission–fusion rate that the amount of repetitive DNA (referred to as heterochromatin) should increase after fission due to the instability of newly generated chromosomes. This would change fresh‐uniarmed chromosomes into biarmed with a near‐fully heterochromatic arm (see Imai, Taylor, and Crozier 1994). In fact, karyotypes of *Acromyrmex* present a higher proportion of heterochromatin revealed by C‐bands scattered across the chromosomes instead of the bands restricted to pericentromeric regions observed in *Atta* and *Amoimyrmex* species (reviewed in Cardoso and Cristiano 2021).

## 5 Conclusions

Even closely related species can exhibit significant differences in their karyotypes (see Cardoso et al. 2014; Cardoso et al. 2018; Moura et al. 2020). The accumulation of these differences can be idiosyncratic and self‐determined. Alternatively, changes may occur in a deterministic manner driven by selection pressures and lineage constraints. The latter case certainly results in a strong phylogenetic signal of karyotype traits, and it certainly applies to karyotype evolution in leafcutting ants. Thus, at different scales, closely related species tend to have more similar karyotype traits compared to less related species. For instance, the presence of telocentric chromosomes is an apomorphy of *Acromyrmex*, whereas the presence of subtelocentric chromosomes is a synapomorphy shared by *Acromyrmex* and *Atta*, while *Amoimyrmex* only harbors submetacentric and metacentric chromosomes. Also, the number of each type of chromosome proportionally increases with the distance from the MRCA in a bimodal fashion. We propose that this situation results from a burst of fissions event producing the major evolutionary transition in the genome architecture of *Acromyrmex*. Therefore, the study reveals that karyotype diversification in leafcutting ants follows a phylogenetic trajectory at differential rates, with differences in karyotype traits reflecting the evolutionary distance between lineages. This finding provides insights into how genetic and chromosomal changes contribute to the evolutionary history and divergence of leafcutting ant species.

## Author Contributions

**Danon Clemes Cardoso:** conceptualization (equal), data curation (equal), formal analysis (equal), funding acquisition (equal), methodology (equal), methodology (equal), resources (equal), resources (equal), software (equal), software (equal), validation (equal), validation (equal), visualization (equal), visualization (equal), writing – original draft (equal), writing – original draft (equal), writing – review and editing (equal), writing – review and editing (equal). **Maykon Passos Cristiano:** conceptualization (equal), data curation (equal), formal analysis (equal), funding acquisition (equal), investigation (equal), methodology (equal), validation (equal), visualization (equal), writing – original draft (equal), writing – review and editing (equal).

## Ethics Statement

The authors have nothing to report.

## Consent

The authors have nothing to report.

## Conflicts of Interest

The authors declare no conflicts of interest.

## Code Availability

Codes used are available by the authors: Puttick, Mark; Ingram, Travis; Clarke, Magnus; Thomas, Gavin (2019). Data from: MOTMOT: Models of trait macroevolution on trees (an update). figshare. Online resource. https://doi.org/10.6084/m9.figshare.11337050.v3 Yoshida and Kitano (2021). Tempo and mode in karyotype evolution revealed by a probabilistic model incorporating both chromosome number and morphology [Dataset]. Dryad. https://doi.org/10.5061/dryad.s4mw6m966.

## Supporting information


Table S1.

## Data Availability

All data has been included in the manuscript. Accessions number for GenBank sequences are given in Supporting Information.

## References

[ece370602-bib-0001] Abouheif, E. 1999. “A Method for Testing the Assumption of Phylogenetic Independence in Comparative Data.” Evolutionary Ecology Research 1: 895–909.

[ece370602-bib-0002] Afonso Neto, P. C. , R. Micolino , D. C. Cardoso , and M. P. Cristiano . 2022. “Phylogenetic Reconstruction of the Ancestral Chromosome Number of the Genera *Anochetus* Mayr, 1861 and *Odontomachus latreille*, 1804 (Hymenoptera: Formicidae: Ponerinae).” Frontiers in Ecology and Evolution 10: 829989. 10.3389/fevo.2022.829989.

[ece370602-bib-0003] Barden, P. 2017. “Fossil Ants (Hymenoptera: Formicidae): Ancient Diversity and the Rise of Modern Lineages.” Myrmecological News 24: 30.

[ece370602-bib-0004] Barros, L. A. , H. J. de Aguiar , S. Mariano Cdos , et al. 2016. “Cytogenetic Data on Six Leafcutter Ants of the Genus *Acromyrmex* Mayr, 1865 (Hymenoptera, Formicidae, Myrmicinae): Insights Into Chromosome Evolution and Taxonomic Implications.” Comparative Cytogenetics 10: 229–243. 10.3897/CompCytogen.v10i2.7612. 27551345 PMC4977799

[ece370602-bib-0005] Barros, L. A. C. , H. J. A. C. Aguiar , G. A. Teixeira , et al. 2015. “Cytogenetic Data on the Threatened Leafcutter Ant *Atta robusta* Borgmeier, 1939 (Formicidae: Myrmicinae: Attini).” Comptes Rendus Biologies 338: 660–665. 10.1016/j.crvi.2015.07.006. 26315727

[ece370602-bib-0006] Barros, L. A. C. , H. J. A. C. Aguiar , G. C. Teixeira , D. J. Souza , J. H. C. Delabie , and C. S. F. Mariano . 2021. “Cytogenetic Studies on the Social Parasite *Acromyrmex Ameliae* (Formicidae: Myrmicinae: Attini) and Its Hosts Reveal Chromosome Fusion in *Acromyrmex* .” Zoologischer Anzeiger 293: 273–281. 10.1016/j.jcz.2021.06.012.

[ece370602-bib-0007] Barros, L. A. C. , G. A. Teixeira , H. J. A. C. Aguiar , C. S. F. Mariano , J. H. C. Delabie , and S. G. Pompolo . 2014. “Banding Patterns of Three Leafcutter Ant Species of the Genus *Atta* (Formicidae: Myrmicinae) and Chromosomal Inferences.” Florida Entomologist 97: 1694–1701. 10.1653/024.097.0444b.

[ece370602-bib-0008] Blomberg, S. P. , T. Garland Jr. , and A. R. Ives . 2003. “Testing for Phylogenetic Signal in Comparative Data: Behavioral Traits Are More Labile.” Evolution 57: 717–745. 10.1111/j.0014-3820.2003.tb00285.x. 12778543

[ece370602-bib-0009] Bouckaert, R. , J. Heled , D. Kühnert , et al. 2014. “BEAST 2: A Software Platform for Bayesian Evolutionary Analysis.” PLoS Computational Biology 10: e1003537. 10.1371/journal.pcbi.1003537. 24722319 PMC3985171

[ece370602-bib-0010] Branstetter, M. G. , A. Ješovnik , J. Sosa‐Calvo , et al. 2017. “Dry Habitats Were Crucibles of Domestication in the Evolution of Agriculture in Ants.” Proceedings of the Biological Sciences 284: 20170095. 10.1098/rspb.2017.0095. PMC5394666 28404776

[ece370602-bib-0011] Cardoso, D. C. , and M. P. Cristiano . 2021. “Karyotype Diversity, Mode, and Tempo of the Chromosomal Evolution of Attina (Formicidae: Myrmicinae: Attini): Is There an Upper Limit to Chromosome Number?” Insects 12: 1084. 10.3390/insects12121084. 34940172 PMC8707115

[ece370602-bib-0012] Cardoso, D. C. , S. das Graças Pompolo , M. P. Cristiano , and M. G. Tavares . 2014. “The Role of Fusion in Ant Chromosome Evolution: Insights From Cytogenetic Analysis Using a Molecular Phylogenetic Approach in the Genus *Mycetophylax* .” PLoS One 9: e87473. 10.1371/journal.pone.0087473. 24489918 PMC3904993

[ece370602-bib-0013] Cardoso, D. C. , J. Heinze , M. N. Moura , and M. P. Cristiano . 2018. “Chromosomal Variation Among Populations of a Fungus‐Farming Ant: Implications for Karyotype Evolution and Potential Restriction to Gene Flow.” BMC Evolutionary Biology 18: 146. 10.1186/s12862-018-1247-5. 30241462 PMC6150965

[ece370602-bib-0014] Cardoso, D. C. , H. G. Santos , and M. P. Cristiano . 2018. “The Ant Chromosome Database–ACdb: An Online Resource for Ant (Hymenoptera: Formicidae) Chromosome Researchers.” Myrmecological News 27: 87–91. 10.25849/myrmecol.news_027:087.

[ece370602-bib-0015] Carta, A. , G. Bedini , and L. Peruzzi . 2018. “Unscrambling Phylogenetic Effects and Ecological Determinants of Chromosome Number in Major Angiosperm Clades.” Scientific Reports 81: 14258. 10.1038/s41598-018-32515-x. PMC6155329 30250220

[ece370602-bib-0016] Castro, C. P. M. , D. C. Cardoso , R. Micolino , and M. P. Cristiano . 2020. “Comparative FISH‐Mapping of TTAGG Telomeric Sequences to the Chromosomes of Leafcutter Ants (Formicidae, Myrmicinae): Is the Insect Canonical Sequence Conserved?” Comparative Cytogenetics 14: 369–385. 10.3897/CompCytogen.v14i3.52726. 32879706 PMC7442751

[ece370602-bib-0017] Cristiano, M. P. , D. C. Cardoso , and T. M. Fernandes‐Salomão . 2013. “Cytogenetic and Molecular Analyses Reveal a Divergence Between *Acromyrmex striatus* (Roger, 1863) and Other Congeneric Species: Taxonomic Implications.” PLoS One 8: e59784. 10.1371/journal.pone.0059784. 23527267 PMC3603875

[ece370602-bib-0072] Cardoso, D. C. , M. N. Moura , and M. P. Cristiano . 2022. “Dynamic development of AT‐rich heterochromatin has followed diversification and genome expansion of psammophilous Mycetophylax (Formicidae: Attini: Attina).” Insect Molecular Biology 31, no. 3: 297–307. Portico. 10.1111/imb.12759. 35060209

[ece370602-bib-0018] Cristiano, M. P. , D. C. Cardoso , V. E. Sandoval‐Gómez , and F. C. Simões‐Gomes . 2020. “ *Amoimyrmex* Cristiano, Cardoso & Sandoval, Gen. Nov. (Hymenoptera: Formicidae): A New Genus of Leaf‐Cutting Ants Revealed by Multilocus Molecular Phylogenetic and Morphological Analyses.” Austral Entomology 59: 643–676. 10.1111/aen.12493.

[ece370602-bib-0019] de Aguiar, H. J. A. C. , L. A. C. Barros , L. I. Silveira , F. Petitclerc , S. Etienne , and J. Orivel . 2020. “Cytogenetic Data for Sixteen Ant Species From North‐Eastern Amazonia With Phylogenetic Insights Into Three Subfamilies.” Comparative Cytogenetics 14: 43–60. 10.3897/CompCytogen.v14i1.46692. 32021662 PMC6989564

[ece370602-bib-0020] Drummond, A. J. , S. Y. W. Ho , M. J. Phillips , and A. Rambaut . 2006. “Relaxed Phylogenetics and Dating With Confidence.” PLoS Biology 4: e88. 10.1371/journal.pbio.0040088. 16683862 PMC1395354

[ece370602-bib-0021] Fadini, M. A. M. , and S. G. Pompolo . 1996. “Cytogenetics of Some Ant Species of the Tribe Attini (Hymenoptera, Formicidae) From the Region of Viçosa.” MG. Revista Brasileira de Genética 19: 53–55.

[ece370602-bib-0022] Faria, R. , and A. Navarro . 2010. “Chromosomal Speciation Revisited: Rearranging Theory With Pieces of Evidence.” Trends in Ecology & Evolution 25: 660–669. 10.1016/j.tree.2010.07.008. 20817305

[ece370602-bib-0023] Felsenstein, J. 1985. “Phylogenies and the Comparative Method.” American Naturalist 125: 1–15. 10.1086/284325.

[ece370602-bib-0024] Glick, L. , and I. Mayrose . 2014. “ChromEvol: Assessing the Pattern of Chromosome Number Evolution and the Inference of Polyploidy Along a Phylogeny.” Molecular Biology and Evolution 31: 1914–1922. 10.1093/molbev/msu122. 24710517

[ece370602-bib-0025] Goñi, B. , L. C. De Zolessi , and H. T. Imai . 1983. “Karyotypes of Thirteen Ant Species From Uruguay (Hymenoptera, Formicidae).” Caryologia 36: 363–371. 10.1080/00087114.1983.10797677.

[ece370602-bib-0026] Harmon, L. J. 2019. “Phylogenetic Comparative Methods: Learning From Trees.” EcoEvoRxiv 1: 234. 10.32942/osf.io/e3xnr.

[ece370602-bib-0027] Harmon, L. J. , J. Weir , C. Brock , R. E. Glor , and W. Challenger . 2008. “GEIGER: Investigating Evolutionary Radiations.” Bioinformatics 24: 129–131. 10.1093/bioinformatics/btm538. 18006550

[ece370602-bib-0028] Heath, T. A. , J. P. Huelsenbeck , and T. Stadler . 2014. “The Fossilized Birth‐Death Process for Coherent Calibration of Divergence‐Time Estimates.” Proc Natl Acad Sci USA 111: e2957–e2966. 10.1073/pnas.1319091111. 25009181 PMC4115571

[ece370602-bib-0029] Imai, H. T. , T. Maruyama , T. Gojobori , Y. Inoue , and R. H. Crozier . 1986. “Theoretical Bases for Karyotype Evolution.” Minimum‐Interaction Hypothesis. American Naturalist 128: 900–920. 10.1086/284612.

[ece370602-bib-0030] Imai, H. T. , Y. Satta , and N. Takahata . 2001. “Integrative Study on Chromosome Evolution of Mammals, Ants and Wasps Based on the Minimum Interaction Theory.” Journal of Theoretical Biology 210: 475–497. 10.1006/jtbi.2001.2327. 11403567

[ece370602-bib-0031] Imai, H. T. , R. W. Taylor , M. W. Crosland , and R. H. Crozier . 1988. “Modes of Spontaneous Chromosomal Mutation and Karyotype Evolution in Ants With Reference to the Minimum Interaction Hypothesis.” Japanese Journal of Genetics 63: 159–185. 10.1266/jjg.63.159. 3273765

[ece370602-bib-0032] Imai, H. T. , R. W. Taylor , and R. H. Crozier . 1994. “Experimental Bases for the Minimum Interaction Theory. I. Chromosome Evolution in Ants of the *Myrmecia pilosula* Species Complex (Hymenoptera: Formicidae: Myrmeciinae).” Japanese Journal of Genetics 69: 137–182. 10.1266/jjg.69.137.

[ece370602-bib-0073] Imai, H. T. , R. H. Crozier , and R. W. Taylor . 1977. “Karyotype evolution in Australian ants.” Chromosoma 59, no. 4: 341–393. 10.1007/bf00327974.

[ece370602-bib-0033] Kandul, N. P. , V. A. Lukhtanov , and N. E. Pierce . 2007. “Karyotypic Diversity and Speciation in *Agrodiaetus* Butterflies.” Evolution 61: 546–559. 10.1111/j.15585646.2007.00046.x. 17348919

[ece370602-bib-0034] King, M. S. 1993. Species Evolution: The Role of Chromosome Change, xxi +336. New York: Cambridge University Press.

[ece370602-bib-0035] Kumar, S. , G. Stecher , and K. Tamura . 2016. “MEGA7: Molecular Evolutionary Genetics Analysis Version 7.0 for Bigger Datasets.” Molecular Biology and Evolution 33: 1870–1874. 10.1093/molbev/msw054. 27004904 PMC8210823

[ece370602-bib-0036] Lanfear, R. , B. Calcott , S. Y. Ho , and S. Guindon . 2012. “Partitionfinder: Combined Selection of Partitioning Schemes and Substitution Models for Phylogenetic Analyses.” Molecular Biology and Evolution 29: 1695–1701. 10.1093/molbev/mss020. 22319168

[ece370602-bib-0037] Lanfear, R. , P. B. Frandsen , A. M. Wright , T. Senfeld , and B. Calcott . 2017. “PartitionFinder 2: New Methods for Selecting Partitioned Models of Evolution for Molecular and Morphological Phylogenetic Analyses.” Molecular Biology and Evolution 34: 772–773. 10.1093/molbev/msw260. 28013191

[ece370602-bib-0038] Levan, A. , K. Fredga , and A. A. Sandberg . 1964. “Nomeclature for Centromeric Position on Chromosomes.” Hereditas 52: 201–220. 10.1111/j.1601-5223.1964.tb01953.x.

[ece370602-bib-0039] Lorite, P. , and T. Palomeque . 2010. “Karyotype Evolution in Ants (Hymenoptera: Formicidae), with a Review of the Known Ant Chromosome Numbers.” Myrmecological News 13: 89–102.

[ece370602-bib-0040] Mackintosh, A. , R. Vila , D. R. Laetsch , A. Hayward , S. H. Martin , and K. Lohse . 2023. “Chromosome Fissions and Fusions Act as Barriers to Gene Flow Between *Brenthis fritillary* Butterflies.” Molecular Biology and Evolution 40: msad043. 10.1093/molbev/msad043. 36810615 PMC10015618

[ece370602-bib-0041] Márquez‐Corro, J. I. , S. Martín‐Bravo , P. Jiménez‐Mejías , et al. 2021. “Macroevolutionary Insights Into Sedges (*Carex*: Cyperaceae): The Effects of Rapid Chromosome Number Evolution on Lineage Diversification.” Journal of Systematics and Evolution 59: 776–790. 10.1111/jse.12730.

[ece370602-bib-0042] Micolino, R. , B. C. L. Baldez , A. F. Sánchez‐Restrepo , L. Calcaterra , M. P. Cristiano , and D. C. Cardoso . 2022. “Karyotype Structure and Cytogenetic Markers of *Amoimyrmex Bruchi* and *Amoimyrmex Silvestrii*: Contribution to Understanding Leaf‐Cutting Ant Relationships.” Genome 65: 43–51. 10.1139/gen-2021-0044. 34520688

[ece370602-bib-0043] Micolino, R. , M. P. Cristiano , and D. C. Cardoso . 2019. “Population‐Based Cytogenetic Banding Analysis and Phylogenetic Relationships of the Neotropical Fungus‐Farming Ant *Trachymyrmex holmgreni* Wheeler, 1925.” Cytogenetic and Genome Research 159: 151–161. 10.1159/000503913. 31683269

[ece370602-bib-0044] Micolino, R. , M. P. Cristiano , and D. C. Cardoso . 2020. “Putative Chromosomal Inversion Clue in the Fungus‐Farming Ant *Mycetomoellerius Iheringi* Emery, 1888.” Comparative Cytogenetics 14: 197–210. 10.3897/CompCytogen.v14i2.49846. 32431788 PMC7225177

[ece370602-bib-0045] Micolino, R. , M. P. Cristiano , N. M. Travenzoli , D. M. Lopes , and D. C. Cardoso . 2019. “Chromosomal Dynamics in Space and Time: Evolutionary History of *Mycetophylax* Ants Across Past Climatic Changes in the Brazilian Atlantic Coast.” Scientific Reports 9: 18800. 10.1038/s41598-019-55135-5. 31827151 PMC6906305

[ece370602-bib-0046] Moran, P. A. P. 1948. “The Interpretation of Statistical Maps.” Journal of the Royal Statistical Society, Series B 10: 243–251.

[ece370602-bib-0047] Moura, M. N. , D. C. Cardoso , and M. P. Cristiano . 2021. “The Tight Genome Size of Ants: Diversity and Evolution Under Ancestral State Reconstruction and Base Composition.” Zoological Journal of the Linnean Society 193: 124–144. 10.1093/zoolinnean/zlaa135.

[ece370602-bib-0048] Moura, M. N. , D. C. Cardoso , B. C. Lima Baldez , and M. P. Cristiano . 2020. “Intraspecific Variation in the Karyotype Length and Genome Size of Fungus‐Farming Ants (Genus *Mycetophylax*), with Remarks on Procedures for the Estimation of Genome Size in the Formicidae by Flow Cytometry.” PLoS One 15: e0237157. 10.1371/journal.pone.0237157. 32760102 PMC7410318

[ece370602-bib-0049] Murakami, T. , A. Fujiwara , and M. C. Yoshida . 1998. “Cytogenetics of Ten Ant Species of the Tribe Attini (Hymenoptera, Formicidae) in Barro Colorado Island, Panama.” Chromosome Science 2: 135–139.

[ece370602-bib-0050] Pagel, M. 1999. “Inferring the Historical Patterns of Biological Evolution.” Nature 401: 877–884. 10553904 10.1038/44766

[ece370602-bib-0051] Paradis, E. , J. Claude , and K. Strimmer . 2004. “APE: Analyses of Phylogenetics and Evolution in R Language.” Bioinformatics 20: 289–290. 14734327 10.1093/bioinformatics/btg412

[ece370602-bib-0052] Pereira, T. T. P. , A. C. C. C. Reis , D. C. Cardoso , and M. P. Cristiano . 2018. “Molecular Phylogenetic Reconstruction and Localization of the (TTAGG)n Telomeric Repeats in the Chromosomes of *Acromyrmex striatus* (Roger, 1863) Suggests a Lower Ancestral Karyotype for Leafcutter Ants (Hymenoptera).” Comparative Cytogenetics 12: 13–26. 10.3897/CompCytogen.v12i1.21799. 29362670 PMC5770561

[ece370602-bib-0053] Puttick, M. N. , T. Ingram , M. Clarke , and G. H. Thomas . 2020. “MOTMOT: Models of Trait Macroevolution on Trees (An Update).” Methods in Ecology and Evolution 11: 464–471. 10.1111/2041-210X.13343.

[ece370602-bib-0054] Puttick, M. N. , G. H. Thomas , and M. J. Benton . 2014. “High Rates of Evolution Preceded the Origin of Birds.” Evolution 68: 1497–1510. 10.1111/evo.12363. 24471891 PMC4289940

[ece370602-bib-0071] R Core Team . 2022. R: A Language and Environment for Statistical Computing. Vienna, Austria: R Foundation for Statistical Computing. https://www.R‐project.org/.

[ece370602-bib-0055] Rambaut, A. 2009. “FigTree v1.4.3.” http://tree.bio.ed.ac.uk/software/figtree.

[ece370602-bib-0070] Rambaut, A. , A. J., Drummond , D. Xie , G. Baele , and M. A. Suchard . 2018. “Posterior Summarization in Bayesian Phylogenetics Using Tracer 1.7.” Systematic Biology 67, no. 5: 901–904. 10.1093/sysbio/syy032. 29718447 PMC6101584

[ece370602-bib-0056] Revell, L. J. 2012. “Phytools: An R Package for Phylogenetic Comparative Biology (and Other Things).” Methods in Ecology and Evolution 3: 217–223. 10.1111/j.2041-210X.2011.00169.x.

[ece370602-bib-0057] Rice, A. , and I. Mayrose . 2020. “Model Adequacy Tests for Probabilistic Models of Chromosome‐Number Evolution.” New Phytologist 229: 3602–3613. 10.1111/nph.17106. 33226654

[ece370602-bib-0058] Rieseberg, L. H. 2001. “Chromosomal Rearrangements and Speciation.” Trends in Ecology & Evolution 16: 351–358. 10.1016/s0169-5347(01)02187-5. 11403867

[ece370602-bib-0059] Ronquist, F. , and J. P. Huelsenbeck . 2003. “MrBayes 3: Bayesian Phylogenetic Inference Under Mixed Models.” Bioinformatics 19: 1572–1574. 10.1093/bioinformatics/btg180. 12912839

[ece370602-bib-0060] Santos‐Colares, M. C. , J. Viégas , M. G. M. Roth , and A. E. Loeck . 1997. “Preparation of Mitotic Chromosomes of Leaf‐Cutting Ants From the Genera *Atta* and Acromyrmex.” Brazilian Journal of Genetics 20: 25–27.

[ece370602-bib-0061] Solomon, S. E. , C. Rabeling , J. Sosa‐Calvo , et al. 2019. “The Molecular Phylogenetics of *Trachymyrmex* Forel Ants and Their Fungal Cultivars Provide Insights Into the Origin and Coevolutionary History of ‘Higher‐Attine’ Ant Agriculture.” Systematic Entomology 44: 939–956. 10.1111/syen.12370.

[ece370602-bib-0062] Teixeira, G. A. , L. A. C. Barros , H. J. A. C. Aguiar , and S. G. Pompolo . 2017. “Comparative Physical Mapping of 18S rDNA in the Karyotypes of Six Leafcutter Ant Species of the Genera *Atta* and *Acromyrmex* (Formicidae: Myrmicinae).” Genetica 145: 351–357. 10.1007/s10709-017-9970-1. 28623426

[ece370602-bib-0063] Thomas, G. H. , and R. P. Freckleton . 2012. “MOTMOT: Models of Trait Macroevolution on Trees.” Methods in Ecology and Evolution 3: 145–151.

[ece370602-bib-0064] Tigano, A. , R. Khan , A. D. Omer , et al. 2022. “Chromosome Size Affects Sequence Divergence Between Species Through the Interplay of Recombination and Selection.” Evolution 76: 782–798. 10.1111/evo.14467. 35271737 PMC9314927

[ece370602-bib-0065] Travenzoli, N. M. , D. C. Cardoso , H. A. Werneck , T. M. Fernandes‐Salomão , M. G. Tavares , and D. M. Lopes . 2019. “The Evolution of Haploid Chromosome Numbers in Meliponini.” PLoS One 14: e0224463. 10.1371/journal.pone.0224463. 31648276 PMC6812824

[ece370602-bib-0066] Vershinina, A. O. , and V. A. Lukhtanov . 2017. “Evolutionary Mechanisms of Runaway Chromosome Number Change in *Agrodiaetus* Butterflies.” Scientific Reports 7: 8199. 10.1038/s41598-017-08525-6. 28811556 PMC5557896

[ece370602-bib-0067] White, M. J. D. 1978. “Modes of Speciation.” W. H. Freeman.

[ece370602-bib-0068] Yoshida, K. , and J. Kitano . 2021. “Tempo and Mode in Karyotype Evolution Revealed by a Probabilistic Model Incorporating Both Chromosome Number and Morphology.” PLoS Genetics 17: e1009502. 10.1371/journal.pgen.1009502. 33861748 PMC8081341

