# Supplementary material for: Shifts in Chromosome Evolution Rates Shape the Karyotype Patterns of Leafcutting Ants
Source: Ecol Evol. 2024 Nov 21;14(11):e70602. doi: 10.1002/ece3.70602 (PMC11582084; doi:10.1002/ece3.70602)
Supplement: Supplementary file 1 — Table S1. [file ECE3-14-e70602-s001.docx]

Supplementary Material

**Table S1**. Ant species used in this study for constructing the multi-locus molecular phylogeny and their accession numbers in GenBank, missing sequences are indicated by “–”.

| **Specimem** | **EF1aF1 exon 1** | **EF1aF1 exon 2** | **EF1aF2** | **opsin exon 1** | **opsin exon 2** | **wingless** | **Top 1** | **mtDNA (COI-trnaLeu-COII** |
| --- | --- | --- | --- | --- | --- | --- | --- | --- |
| *Acromyrmex ambiguus* (2) | KC478109 | | KC478126 | JX198244 | JX198244 | JX198232 | - | - |
| *Acromyrmex ameliae* (ACMA0017) | MT625672 | | MT625538 | MT625583 | MT625628 | MT625494 | - | - |
| *Acromyrmex aspersus* (ACSP0108) | MT625664 | | MT625530 | MT625575 | MT625620 | MT625487 | - | - |
| *Acromyrmex balzani* | EU204323 | EU204414 | EU204565 | EU204490 | EU204247 | EU204170 | KY828557 | AY265964 |
| *Acromyrmex coronatus* (ACCR0127) | MT625677 | | MT625543 | MT625588 | MT625633 | MT625499 | - | AY265969 |
| *Acromyrmex crassispinus*  (ACSB0262) | MT625670 | | MT625536 | MT625581 | MT625626 | MT625492 | - | - |
| *Acromyrmex crassispinus*  (ACSP0106) | MT625665 | | MT625531 | MT625576 | MT625621 | MT625488 | - | AY265970 |
| *Acromyrmex disciger* (ACDS0125) | MT625676 | | MT625542 | MT625587 | MT625632 | MT625498 | - | - |
| *Acromyrmex echinatior* | KC478113 | | KC478130 | KC478104 | KC478104 | KC478095 | - | AY265963 |
| *Acromyrmex fracticornis* (ACFR0003) | MT625675 | | MT625541 | MT625586 | MT625631 | MT625497 | - | - |
| *Acromyrmex heyeri* | EU204363 | EU204453 | EU204604 | EU204529 | EU204286 | EU204210 | KY828555 | AY265971 |
| *Acromyrmex hispidus* (ACHI0009) | MT625674 | | MT625540 | MT625585 | MT625630 | MT625496 | - | - |
| *Acromyrmex hispidus fallax* (ACSP0052) | MT625668 | | MT625534 | MT625579 | MT625624 | - | - | - |
| *Acromyrmex insinuator* (2) | – | – | – | – | – | – | – | MK241586 |
| *Acromyrmex landolti* | EU204364 | EU204454 | EU204605 | EU204530 | EU204287 | EU204211 | KY828558 | - |
| *Acromyrmex laticeps* (ACLA0001) | MT625673 | | MT625539 | MT625584 | MT625629 | MT625495 | - | - |
| *Acromyrmex lobicornis* | – | – | – | – | – | – | – | MG753550 |
| *Acromyrmex lundi* (3) | EU204361 | EU204451 | EU204602 | EU204527 | EU204284 | EU204208 | KY828553 | AY265967 |
| *Acromyrmex niger* (ACNG0123) | MT625671 | | MT625537 | MT625582 | MT625627 | MT625493 | - | - |
| *Acromyrmex nigrosetosus* (ACSP0214) | MT625662 | | MT625528 | MT625573 | MT625618 | MT625485 | - | - |
| *Acromyrmex octospinosus* | EU204298 | EU204389 | EU204541 | EU204465 | EU204222 | EU204145 | KY828554 | AY265965 |
| *Acromyrmex rugosus* (ACSP0062) | MT625667 | | MT625533 | MT625578 | MT625623 | MT625490 | - | AY265968 |
| *Acromyrmex striatus* | MK600307 | | MK599980 | MK600122 | | MK600200 | KY828501 | - |
| *Acromyrmex striatus* | – | – | – | – | – | – | – | KR605606 |
| *Acromyrmex subterraneus molestans* (ACSM0122) | MT625669 | | MT625535 | MT625580 | MT625625 | MT625491 | - | - |
| *Acromyrmex subterraneus subterraneus* (ACSP0126) | MT625663 | | MT625529 | MT625574 | MT625619 | MT625486 | - | AY265962 |
| *Acromyrmex subterranus* (ACSP0083) | MT625666 | | MT625532 | MT625577 | MT625622 | MT625489 | - | - |
| *Acromyrmex versicolor* | EU204378 | EF013211 | EF013373 | EF013534 | EF013534 | EF013662 | KJ861521 | - |
| *Acromyrmex volcanus* | – | – | – | – | – | – | – | MK207450 |
| *Amoimyrmex bruchii* (ACST0079) | MT625661 | | MT625527 | MT625572 | MT625617 | MT625484 | MT625455 | MT627327 |
| *Amoimyrmex silveestrii* (ACST0081) | MT625659 | | MT625525 | MT625570 | MT625615 | MT625482 | MT625454 | KR605629 |
| *Amoimyrmex striatus* (ACST0114) | MT625658 | | MT625524 | MT625569 | MT625614 | MT625481 | MT625453 | KR605606 |
| *Apterostigma auriculatum* 1 | EU204377 | EF013230 | EF013392 | EF013549 | EF013549 | EF013677 | - | - |
| *Apterostigma collare* | EU204374 | EU204464 | EU204615 | EU204540 | EU204297 | EU204221 | KY828524 | - |
| *Apterostigma dentigerum* | EU204349 | EU204440 | EU204590 | EU204515 | EU204272 | EU204196 | - | - |
| *Apterostigma dorotheae* | EU204334 | EU204425 | EU204576 | EU204500 | EU204257 | EU204181 | KY828520 | - |
| *Apterostigma goniodes* | EU204347 | EU204438 | EU204588 | EU204513 | EU204270 | EU204194 | KY828523 | - |
| *Apterostigma manni* | EU204318 | EU204409 | EU204560 | EU204485 | EU204242 | EU204165 | KY828521 | - |
| *Apterostigma megacephala* | KP406345 | KP406346 | KP406350 | KP406348 | KP406349 | KP406347 | KY828518 | - |
| *Apterostigma pilosum cplx* sp.4 | EU204348 | EU204439 | EU204589 | EU204514 | EU204271 | EU204195 | KY828525 | - |
| *Atta bisphaerica* | KC478121 | | KC478138 | KC478102 | KC478102 | KC478098 | - | FJ547455 |
| *Atta capiguara* | – | – | – | – | – | – | – | FJ547449 |
| *Atta cephalotes* | EU204350 | EU204441 | EU204591 | EU204516 | EU204273 | EU204197 | KY828559 | EU156273 |
| *Atta colombica* | KC478122 | | KC478139 | KC478103 | KC478103 | KC478099 | - | EU848060 |
| *Atta insularis* | – | – | – | – | – | – | – | EU165431 |
| *Atta laevigata* | EU204314 | EU204405 | EU204556 | EU204481 | EU204238 | EU204161 | KY828561 | EU848122 |
| *Atta mexicana* | EU204324 | EU204415 | EU204566 | EU204491 | EU204248 | EU204171 | KY828560 | EU165430 |
| *Atta opacipes* (ATSP0109) | MT625634 | | MT625500 | MT625545 | MT625590 | MT625457 | - | FJ547470 |
| *Atta robusta* | KC478123 | | KC478140 | JX198254 | JX198254 | JX198242 | - | FJ547444 |
| *Atta saltensis* | – | – | – | – | – | – | – | FJ547469 |
| *Atta sexdens rubropilosa* | KC478125 | | KC478142 | KC478106 | KC478106 | KC478101 | - | AF016018 |
| *Atta texana* | EU204359 | EU204449 | EU204600 | EU204525 | EU204282 | EU204206 | KY828562 | EU848062 |
| *Atta vollenweideri* | – | – | – | – | – | – | – | FJ547453.1 |
| *Basiceros manni* | EU204379 | EF013232 | EF013394 | EF013551 | EF013551 | EF013679 | KJ861542 | - |
| *Blepharidatta brasiliensis* | EU204315 | EU204406 | EU204557 | EU204482 | EU204239 | EU204162 | KY828506 | - |
| *Cataulacus ebrardi* | EU204380 | EF013240 | EF013402 | EF013558 | EF013558 | EF013686 | KM578900 | - |
| *Cephalotes atratus* | EU204313 | EU204404 | EU204555 | EU204480 | EU204237 | EU204160 | KY828505 | - |
| *Crematogaster sp.* | EU204328 | EU204419 | EU204570 | EU204494 | EU204251 | EU204175 | - | - |
| *Cyatta abscondita* | KF569882 | KF569883 | KF569884. | KF569885 | KF569886 | KF569887 | KY828527 | - |
| *Cyphomyrmex cornutus* | EU204355 | EU204445 | EU204596 | EU204521 | EU204278 | EU204202 | KY828533 | - |
| *Cyphomyrmex cornutus* 1 | EU204366 | EU204456 | EU204607 | EU204532 | EU204289 | EU204213 | - | - |
| *Cyphomyrmex costatus* | EU204321 | EU204412 | EU204563 | EU204488 | EU204245 | EU204168 | KY828535 | - |
| *Cyphomyrmex longiscapus* (Fortuna) | EU204368 | EU204458 | EU204609 | EU204534 | EU204291 | EU204215 | KY828541 | - |
| *Cyphomyrmex longiscapus* (SS) | EU204330 | EU204421 | EU204572 | EU204496 | EU204253 | EU204177 | KY828536 | - |
| *Cyphomyrmex minutus* | EU204342 | EU204433 | EU204583 | EU204508 | EU204265 | EU204189 | KY828537 | - |
| *Cyphomyrmex muelleri* | EU204369 | EU204459 | EU204610 | EU204535 | EU204292 | EU204216 | KY828542 | - |
| *Cyphomyrmex nsp. Brazil* | EU204354 | - | EU204595 | EU204520 | EU204277 | EU204201 | KY828538 | - |
| *Cyphomyrmex rimosus* | EU204299 | EU204390 | MK599982 | EU204466 | EU204223 | EU204146 | KY828540 | - |
| *Cyphomyrmex strigatus* (Gr) | KY828579 | | KY828485 | KY828589 | | KY828495 | KY828499 | - |
| *Cyphomyrmex strigatus* (Gr1) | KY828578 | | KY828484 | KY828588 | | KY828494 | KY828498 | - |
| *Cyphomyrmex strigatus* (Gr2) | KY828576 | | KY828482 | KY828586 | | KY828492 | KY828568 | - |
| *Cyphomyrmex strigatus* (Gr3) | KY828575 | | KY828481 | KY828585 | | KY828491 | KY828567 | - |
| *Cyphomyrmex strigatus* (Grp) | KY828573 | | KY828480 | KY828583 | | KY828489 | KY828565 | - |
| *Cyphomyrmex strigatus* (Grp1) | KY828572 | | KY828479 | KY828582 | | KY828488 | KY828564 | - |
| *Daceton armigerum* | EU204376 | EF013251 | EF013414 | EF013565 | EF013565 | EF013693 | KJ861572 | - |
| *Kalathomyrmex emeryi* | EU204358 | EU204448 | EU204599 | EU204524 | EU204281 | EU204205 | KY828528 | - |
| *Kalathomyrmex emeryi* 1 | EU204311 | EU204402 | EU204553 | EU204478 | EU204235 | EU204158 | - | - |
| *Meranoplus sp.* | EU204346 | EU204437 | EU204587 | EU204512 | EU204269 | EU204193 | - | - |
| *Monomorium pharaonis* | EU204326 | EU204417 | EU204568 | - | - | EU204173 | KY828508 | - |
| *Mycetagroicus cerradensis* | MK600309 | | MK599983 | MK600124 | | MK600215 | KY828500 | - |
| *Mycetagroicus triangularis* | EU204371 | EU204461 | EU204612 | EU204537 | EU204294 | EU204218 | - | - |
| *Mycetarotes acutus* | EU204351 | EU204442 | EU204592 | EU204517 | EU204274 | EU204198 | KY828526 | - |
| *Mycetarotes parallelus* | EU204307 | EU204398 | EU204549 | EU204474 | EU204231 | EU204154 | - | - |
| *Mycetomoellerius atlanticus* | MK600312 | | MK599986 | MK600127 | | MK600220 | – | MK600060 |
| *Mycetomoellerius cirratus* | MK600313 | | MK599987 | MK600128 | | MK600221 | – | MK600061 |
| *Mycetomoellerius dichrous* | MK600314 | | MK599988 | MK600129 | | MK600222 | – | MK600062 |
| *Mycetomoellerius fuscus* | MK600317 | | MK599991 | MK600132 | | MK600225 | – | MK600065 |
| *Mycetomoellerius holmgreni* | MK600319 | | MK599993 | MK600134 | | MK600227 | – | MK600067 |
| *Mycetomoellerius kempfi* | MK600324 | | MK599998 | MK600139 | | MK600232 | – | MK600071 |
| *Mycetomoellerius nsp* | EU204333 | | EU204575 | EU204499 | | MK600238 | – | – |
| *Mycetomoellerius nsp* | MK600333 | | MK600007 | MK600148 | | MK600243 | – | MK600080 |
| *Mycetomoellerius nsp* | MK600334 | | MK600008 | MK600149 | | MK600244 | – | MK600081 |
| *Mycetomoellerius nsp nr cirratus* | MK600336 | | MK600010 | MK600151 | | MK600246 | – | – |
| *Mycetomoellerius nsp nr dichrous* | MK600337 | | MK600011 | MK600152 | | MK600247 | – | MK600083 |
| *Mycetomoellerius nsp nr haytianus* | MK600338 | | MK600012 | MK600153 | | MK600248 | – | MK600084 |
| *Mycetomoellerius nsp1 nr atlanticus* | MK600328 | | MK600002 | MK600143 | | MK600236 | – | MK600075 |
| *Mycetomoellerius nsp2 nr atlanticus* | MK600329 | | MK600003 | MK600144 | | MK600237 | – | MK600076 |
| *Mycetomoellerius opulentus* | MK600339 | | MK600013 | MK600154 | | MK600250 | – | MK600085 |
| *Mycetomoellerius papulatus* | EU204338 | | EU204579 | EU204504 | | MK600251 | – | MK600086 |
| *Mycetomoellerius relictus* | MK600340 | | MK600014 | MK600155 | | MK600252 | – | MK600087 |
| *Mycetomoellerius zeteki* | MK600342 | | MK600016 | MK600157 | | MK600253 | – | MK600089 |
| *Mycetophylax andersoni* | KY828574 | | - | KY828584 | | KY828490 | KY828563 | - |
| *Mycetophylax asper* | KY828577 | | KY828483 | KY828587 | | KY828493 | KY828566 | - |
| *Mycetophylax clorindae* | EU204370 | EU204460 | EU204611 | EU204536 | EU204293 | EU204217 | KY828543 | - |
| *Mycetophylax conformis* | EU204319 | EU204410 | EU204561 | EU204486 | EU204243 | EU204166 | KY828530 | - |
| *Mycetophylax faunulus* | EU204320 | EU204411 | EU204562 | EU204487 | EU204244 | EU204167 | KY828534 | - |
| *Mycetophylax morschi - 148* | MN745227 | | MN745257 | MN745257 | | MN745212 | MN745272 | - |
| *Mycetophylax morschi - 191* | MN745237 | | MN745267 | MN745252 | | MN745222 | MN745282 | - |
| *Mycetophylax morschi -179* | MN745232 | | MN745262 | MN745247 | | MN745217 | MN745277 | - |
| *Mycetophylax simplex* |  |  |  | KC964627 | |  | KC964649 | - |
| *Mycetosoritis hartmanni* | EU204312 | EU204403 | EU204554 | EU204479 | EU204236 | EU204159 | KY828529 | - |
| *Mycocepurus curvispinosus* | EU204343 | EU204434 | EU204584 | EU204509 | EU204266 | EU204190 | KY828516 | - |
| *Mycocepurus smithii 1* | EU204310 | EU204401 | EU204552 | EU204477 | EU204234 | EU204157 | - | - |
| *Mycocepurus smithii 2* | EU204357 | EU204447 | EU204598 | EU204523 | EU204280 | EU204204 | KY828517 | - |
| *Mycocepurus tardus* | EU204341 | EU204432 | EU204582 | EU204507 | EU204264 | EU204188 | KY828515 | - |
| *Myrmica sp.* | EU204305 | EU204396 | EU204547 | EU204472 | EU204229 | EU204152 | - | - |
| *Myrmica striolagaster* | EU204381 | EF013296 | EF013458 | EF013598 | EF013598 | EF013726 | KJ861639 | - |
| *Myrmicocrypta buenzlii* | EU204344 | EU204435 | EU204585 | EU204510 | EU204267 | EU204191 | KY828513 | - |
| *Myrmicocrypta camargoi* | EU204356 | EU204446 | EU204597 | EU204522 | EU204279 | EU204203 | KY828512 | - |
| *Myrmicocrypta ednaella* | EU204373 | EU204463 | EU204614 | EU204539 | EU204296 | EU204220 | KY828510 | - |
| *Myrmicocrypta infuscata* | EU204375 | EF013299 | EF013461 | EF013600 | EF013600 | EF013728 | KY828511 | - |
| *Myrmicocrypta sp.1 Panama* | EU204340 | EU204431 | EU204581 | EU204506 | EU204263 | EU204187 | KY828514 | - |
| *Myrmicocrypta urichi* | EU204304 | EU204395 | EU204546 | EU204471 | EU204228 | EU204151 | KY828509 | - |
| *Orectognathus sp.* | EU204352 | EU204443 | EU204593 | EU204518 | EU204275 | EU204199 | - | - |
| *Orectognathus versicolor* | EU204382 | EF013312 | EF013474 | EF013611 | EF013611 | EF013739 | KJ861656 | - |
| *Paratrachymyrmex bugnioni* | MK600344 | | MK600018 | MK600159 | | MK600259 | – | MK600090 |
| *Paratrachymyrmex cf carib* | MK600345 | | MK600019 | MK600160 | | MK600261 | – | MK600091 |
| *Paratrachymyrmex cf irmgardae* | MK600346 | | MK600020 | MK600161 | | MK600262 | – | MK600092 |
| *Paratrachymyrmex cornetzi* | MK600348 | | MK600022 | MK600163 | | MK600265 | – | MK600094 |
| *Paratrachymyrmex diversus* | MK600352 | | MK600026 | MK600167 | | MK600270 | – | MK600098 |
| *Paratrachymyrmex intermedius* | MK600353 | | MK600028 | MK600168 | | MK600272 | – | MK600099 |
| *Paratrachymyrmex nsp nr bugnioni* | MK600354 | | MK600029 | MK600169 | | MK600273 | – | MK600100 |
| *Paratrachymyrmex nsp nr cornetzi* | MK600356 | | MK600031 | MK600171 | | MK600275 | – | MK600102 |
| *Pheidole clydei* | EU204383 | EF013317 | EF013479 | EF013615 | EF013615 | EF013743 | KJ861662 | - |
| *Pheidole hyatti* | EU204384 | EF013318 | EF013480 | EF013616 | EF013616 | EF013744 | KJ861664 | - |
| *Pogonomyrmex sp.* | EU204325 | EU204416 | EU204567 | EU204492 | EU204249 | EU204172 | KY828502 | - |
| *Pristomyrmex pungens* | EU204353 | EU204444 | EU204594 | EU204519 | EU204276 | EU204200 | - | - |
| *Proatta butteli* | EU204329 | EU204420 | EU204571 | EU204495 | EU204252 | EU204176 | - | - |
| *Procryptocerus scabriusculus* | EU204385 | EF013336 | EF013498 | EF013632 | EF013632 | EF013760 | KJ861680 | - |
| *Pseudoatta nsp.* | EU204327 | EU204418 | EU204569 | EU204493 | EU204250 | EU204174 | KY828556 | - |
| *Pseudoatta sp.* SS-200 | – | – | – | – | – | – | – | AY265972 |
| *Sericomyrmex bondari* | MK600359 | | MK600034 | MK600173 | | MK600279 | – | – |
| *Sericomyrmex parvulus* | EU204300 | EU204391 | EU204542 | EU204467 | EU204224 | EU204147 | KY828531 | - |
| *Sericomyrmex parvulus* | EU204300 | | EU204542 | EU204467 | | MK600280 | – | – |
| *Sericomyrmex parvulus* | MK600360 | | MK600035 | MK600174 | | MK600281 | – | – |
| *Sericomyrmex saussurei* | MK600361 | | MK600036 | – | | MK600282 | – | – |
| *Sericomyrmex sp* | MK600362 | | MK600037 | MK600175 | | MK600283 | – | MK600105 |
| *Sericomyrmex sp.* (938 Peru) | KY828581 | | KY828487 | KY828592 | | KY828497 | KY828570 | - |
| *Sericomyrmex sp.* (944 Guyana) | KY828580 | | KY828486 | KY828591 | | KY828496 | KY828569 | - |
| *Strumigenys dicomas* | EU204387 | EF013352 | EF013514 | EF013645 | EF013645 | EF013773 | KJ861712 | - |
| *Strumigenys exiguaevitae* | EU204386 | EF013341 | EF013503 | EF013636 | EF013636 | EF013764 | KJ861715 | - |
| *Strumigenys propiciens* | EU204306 | EU204397 | EU204548 | EU204473 | EU204230 | EU204153 | KY828503 | - |
| *Tetramorium caespitum* | EU204308 | EU204399 | EU204550 | EU204475 | EU204232 | EU204155 | - | - |
| *Trachymyrmex arizonensis* | EU204388 | EF013364 | EF013526 | EF013655 | EF013655 | EF013783 | KJ861741 | MK600107 |
| *Trachymyrmex arizonensis* | MK600364 | | MK600039 | MK600177 | | MK600285 | – | MK600107 |
| *Trachymyrmex bugnioni* | EU204303 | EU204394 | EU204545 | EU204470 | EU204227 | EU204150 | KY828550 | - |
| *Trachymyrmex carinatus* | MK600366 | | MK600041 | MK600179 | | MK600288 | – | MK600109 |
| *Trachymyrmex cornetzi* | EU204301 | EU204392 | EU204543 | EU204468 | EU204225 | EU204148 | KY828548 | - |
| *Trachymyrmex desertorum* | MK600368 | | MK600043 | MK600181 | | MK600290 | – | MK600111 |
| *Trachymyrmex diversus* | EU204302 | EU204393 | EU204544 | EU204469 | EU204226 | EU204149 | KY828549 | - |
| *Trachymyrmex intermedius* | EU204336 | EU204427 | MK600027 | EU204502 | EU204259 | EU204183 | KY828544 | - |
| *Trachymyrmex irmgardae* | EU204322 | EU204413 | EU204564 | EU204489 | EU204246 | EU204169 | KY828546 | - |
| *Trachymyrmex jamaicensis* | MK600369 | | MK600044 | MK600182 | | MK600291 | – | MK600112 |
| *Trachymyrmex nogalensis* | MK600371 | | MK600046 | MK600184 | | MK600293 | – | – |
| *Trachymyrmex nsp.* | EU204333 | EU204424 | EU204575 | EU204499 | EU204256 | EU204180 | KY828532 | - |
| *Trachymyrmex opulentus* | EU204332 | EU204423 | EU204574 | EU204498 | EU204255 | EU204179 | KY828547 | - |
| *Trachymyrmex papulatus* | EU204338 | EU204429 | EU204579 | EU204504 | EU204261 | EU204185 | KY828545 | - |
| *Trachymyrmex pomonae* | MK600373 | | MK600048 | MK600186 | | MK600295 | – | MK600113 |
| *Trachymyrmex ruthae* | MK600374 | | MK600049 | MK600187 | | MK600296 | – | MK600114 |
| *Trachymyrmex saussurei* | MK600341 | | MK600015 | MK600156 | | MK600297 | – | MK600088 |
| *Trachymyrmex septentrionalis* | EU204337 | EU204428 | EU204578 | EU204503 | EU204260 | EU204184 | KY828551 | KP283014 |
| *Trachymyrmex septentrionalis* | MK600376 | | MK600051 | MK600189 | | MK600300 | – | MK600117 |
| *Trachymyrmex smithi* | EU204372 | EU204462 | EU204613 | EU204538 | EU204295 | EU204219 | KY828552 | - |
| *Trachymyrmex smithi* | MK600378 | | MK600053 | MK600191 | | MK600303 | – | MK600119 |
| *Trachymyrmex turrifex* | MK600379 | | MK600054 | MK600192 | | MK600304 | – | MK600120 |
| *Trachymyrmex urichii* | MK600381 | | MK600056 | MK600194 | | MK600306 | – | MK600121 |
| *Trachymyrmex zeteki* | EU204339 | EU204430 | EU204580 | EU204505 | EU204262 | EU204186 | - | - |
| *Tranopelta gilva* | EU204309 | EU204400 | EU204551 | EU204476 | EU204233 | EU204156 | KY828504 | - |
| *Wasmannia auropunctata* | EU204316 | EU204407 | EU204558 | EU204483 | EU204240 | EU204163 | - | - |
| *Wasmannia auropunctata* | EU204362 | EU204452 | EU204603 | EU204528 | EU204285 | EU204209 | KY828507 | - |
| *Xerolitor* *explicatus* | MG642983 | | MG642985 | MG642986 | | MG642985 | MG642987 | – |

**Table S2.** Models of molecular evolution by genes and codons implemented in the Bayesian analyses to infer the phylogenetic hypothesis of leafcutting ants implemented in the analysis of the study. Genes:

| **Evolutionary model** | | **# sites** | **Partitions** |
| --- | --- | --- | --- |
| **1.** | TRN+I | 1086 | WG_pos1, WG_pos2, EF1aF1_pos1, TOP1_pos1, EF1aF2_pos1 |
| **2.** | HKY+I | 966 | LwRH_pos1, TOP1_pos2, EF1aF2_pos2, EF1aF1_pos2 |
| **3.** | TRN+G | 818 | WG_pos3, EF1aF1_pos3, EF1aF2_pos3, LwRh_pos3 |
| **4.** | F81+I | 653 | LwRh_pos2, coi_pos2 |
| **5.** | HKY+I | 284 | TOP1_pos3 |
| **6.** | GTR+G | 775 | tRNAleu, coii_pos1, coii_pos2, coi_pos1 |
| **7.** | TIM+G | 598 | coi_pos3, coii_pos3 |

Genes: *wingless* (WG), *elongation factor-1 alpha* F1 (EF1aF1), *elongation factor-1 alpha* F2 (EF1aF2), *long-wavelength rhodopsin* (LwRh),

pos1: 1st – first position

pos2: 2nd – second position

pos3: 3rd – third position
